# Global, regional, and national burden of pulmonary arterial hypertension, 1990–2021: a systematic analysis for the Global Burden of Disease Study 2021

**DOI:** 10.1016/S2213-2600(24)00295-9

**Published:** 2025-01

**Authors:** GBD 2021 Pulmonary Arterial Hypertension Collaborators, Peter J Leary, Megan Lindstrom, Catherine O Johnson, Sophia Emmons-Bell, Stuart Rich, Paul A Corris, Hilary M DuBrock, Corey E Ventetuolo, Yohannes Habtegiorgis Abate, Michael Abdelmasseh, Richard Gyan Aboagye, Hasan Abualruz, Eman Abu-Gharbieh, Salahdein Aburuz, Lawan Hassan Adamu, Rui Adão, Isaac Yeboah Addo, Rufus Adesoji Adedoyin, Juliana Bunmi Adetunji, Leticia Akua Adzigbli, Bright Opoku Ahinkorah, Firdos Ahmad, Amir Mahmoud Ahmadzade, Ayman Ahmed, Haroon Ahmed, Syed Anees Ahmed, Shiva Akhlaghi, Mohammed Ahmed Akkaif, Salah Al Awaidy, Samer O Alalalmeh, Almaza Albakri, Khalifah A Aldawsari, Wael Almahmeed, Najim Z Alshahrani, Awais Altaf, Hany Aly, Karem H Alzoubi, Walid Adnan Al-Zyoud, Reza Amani, Ganiyu Adeniyi Amusa, Catalina Liliana Andrei, Saleha Anwar, Anayochukwu Edward Anyasodor, Aleksandr Y Aravkin, Demelash Areda, Haftu Asmerom Asmerom, Avinash Aujayeb, Ahmed Y. Azzam, Abraham Samuel Babu, Sara Bagherieh, Ovidiu Constantin Baltatu, Hiba Jawdat Barqawi, Mohammad-Mahdi Bastan, Kavita Batra, Nebiyou Simegnew Bayleyegn, Amir Hossein Behnoush, Jaideep Singh Bhalla, Sonu Bhaskar, Vivek Bhat, Saeid Bitaraf, Veera R Bitra, Archith Boloor, Dejana Braithwaite, Michael Brauer, Lemma N Bulto, Yasser Bustanji, Vijay Kumar Chattu, Gerald Chi, Fatemeh Chichagi, Bryan Chong, Rajiv Chowdhury, Zinhle Cindi, Natalia Cruz-Martins, Sriharsha Dadana, Omid Dadras, Tukur Dahiru, Xiaochen Dai, Mohadese Dashtkoohi, Sean DeAngelo, Shayom Debopadhaya, Berecha Hundessa Demessa, Hardik Dineshbhai Desai, Vishal R Dhulipala, Michael J Diaz, Mengistie Diress, Thanh Chi Do, Thao Huynh Phuong Do, Khanh Duy Doan, Wendel Mombaque dos Santos, Rajkumar Prakashbhai Doshi, Robert Kokou Dowou, Arkadiusz Marian Dziedzic, Muhammed Elhadi, Farshid Etaee, Natalia Fabin, Adeniyi Francis Fagbamigbe, Pawan Sirwan Faris, Bikila Regassa Feyisa, Celia Fortuna Rodrigues, Aravind P Gandhi, Mohammad Arfat Ganiyani, Yibeltal Yismaw Gela, Molla Getie, Amir Ghaffari Jolfayi, Afsaneh Ghasemzadeh, Mohamad Goldust, Mahaveer Golechha, Shi-Yang Guan, Mesay Dechasa Gudeta, Mohak Gupta, Rahul Gupta, Mostafa Hadei, Ahmad Hammoud, Md Saquib Hasnain, Mahgol Sadat Hassan Zadeh Tabatabaei, Simon I Hay, Omar E Hegazi, Mehdi Hemmati, Yuta Hiraike, Nguyen Quoc Hoan, Michael Hultström, Hong-Han Huynh, Segun Emmanuel Ibitoye, Olayinka Stephen Ilesanmi, Nahlah Elkudssiah Ismail, Chidozie Declan Iwu, Khushleen Jaggi, Akhil Jain, Mihajlo Jakovljevic, Sun Ha Jee, Bijay Mukesh Jeswani, Anil K Jha, Mohammad Jokar, Nitin Joseph, Jacek Jerzy Jozwiak, Hannaneh Kabir, Farima Kahe, Arun Kamireddy, Arun R Kanmanthareddy, Hanie Karimi, Arman Karimi Behnagh, Sina Kazemian, Pedram Keshavarz, Amirmohammad Khalaji, Mohammad Jobair Khan, Feriha Fatima Khidri, Min Seo Kim, Shivakumar KM Marulasiddaiah Kondlahalli, Nikhil Kothari, Kewal Krishan, Mukhtar Kulimbet, Ashish Kumar, Kaveh Latifinaibin, Thao Thi Thu Le, Caterina Ledda, Seung Won Lee, Ming-Chieh Li, Stephen S Lim, Shuke Liu, Elham Mahmoudi, Omar M Makram, Kashish Malhotra, Ahmad Azam Malik, Deborah Carvalho Malta, Yosef Manla, Miquel Martorell, Kamran Mehrabani-Zeinabad, Mohsen Merati, Tomislav Mestrovic, Niloofar Mirdamadi, Arup Kumar Misra, Ali H Mokdad, Mohammad Ali Moni, AmirAli Moodi Ghalibaf, Paula Moraga, Negar Morovatdar, Rohith Motappa, Seyed Ali Mousavi-Aghdas, Ahmad Mustafa, Ganesh R Naik, Mohammad Sadeq Najafi, Soroush Najdaghi, Dhairya P Nanavaty, Delaram Narimani Davani, Zuhair S Natto, Javaid Nauman, Dang H Nguyen, Phat Tuan Nguyen, Robina Khan Niazi, Bogdan Oancea, Titilope O Olanipekun, Gláucia Maria Moraes Oliveira, Hany A Omar, Mahesh Padukudru P A, Feng Pan, Seithikurippu R Pandi-Perumal, Ioannis Pantazopoulos, Romil R Parikh, Ionela-Roxana Petcu, Hoang Nhat Pham, Hoang Tran Pham, Anil K Philip, Elton Junio Sady Prates, Jagadeesh Puvvula, Gangzhen Qian, Quinn Rafferty, Fakher Rahim, Mehran Rahimi, Mosiur Rahman, Muhammad Aziz Rahman, Mohammad Rahmanian, Nazanin Rahmanian, Masoud Rahmati, Rahem Rahmati, Mahmoud Mohammed Ramadan, Kamleshun Ramphul, Juwel Rana, Indu Ramachandra Rao, Sina Rashedi, Nakul Ravikumar, Salman Rawaf, Ayita Ray, Murali Mohan Rama Krishna Reddy, Elrashdy Moustafa Mohamed Redwan, Negar Rezaei, Priyanka Roy, Aly M A Saad, Basema Ahmad Saddik, Masoumeh Sadeghi, Mohammad Reza Saeb, Fatemeh Saheb Sharif-Askari, Narjes Saheb Sharif-Askari, Mohamed A Saleh, Najib Yahaya Sani, Ushasi Saraswati, Aswini Saravanan, Jennifer Saulam, Art Schuermans, Austin E Schumacher, Birhan Ewunu Semagn, Yashendra Sethi, Allen Seylani, Melika Shafeghat, Moyad Jamal Shahwan, Muhammad Aaqib Shamim, Anas Shamsi, Sadaf Sharfaei, Kamal Sharma, Nitish Sharma, Akil Adrian Sherif, Ivy Shiue, Seyed Afshin Shorofi, Emmanuel Edwar Siddig, Harpreet Singh, Jasvinder A Singh, Paramdeep Singh, Surjit Singh, Farrukh Sobia, Ranjan Solanki, Shipra Solanki, Michael Spartalis, Chandan Kumar Swain, Lukasz Szarpak, Seyyed Mohammad Tabatabaei, Celine Tabche, Jacques Lukenze Tamuzi, Ker-Kan Tan, Masayuki Teramoto, Samar Tharwat, Friedrich Thienemann, Thien Tan Tri Tai Truyen, Guesh Mebrahtom Tsegay, Aniefiok John Udoakang, Jef Van den Eynde, Shoban Babu Varthya, Madhur Verma, Dominique Vervoort, Manish Vinayak, Maria Viskadourou, Fang Wang, Nuwan Darshana Wickramasinghe, Angga Wilandika, Suowen Xu, Chuanhua Yu, Iman Zare, Mohammad A Zeineddine, Zhi-Jiang Zhang, Lei Zhu, Abzal Zhumagaliuly, Magdalena Zielińska, Samer H Zyoud, Christopher J L Murray, Gregory A Roth

## Abstract

**Background:**

Pulmonary arterial hypertension (PAH) is a vascular disease characterised by restricted flow and high pressure through the pulmonary arteries, leading to progressive right heart failure and death. This study reports the global burden of PAH, leveraging all available data and using methodology of the Global Burden of Diseases, Injuries, and Risk Factors Study (GBD) to understand the epidemiology of this under-researched and morbid disease.

**Methods:**

Prior to the current effort, the burden of PAH was included in GBD as a non-specific contributor to “other cardiovascular and circulatory disease” burden. In this study, PAH was distinguished as its own cause of death and disability in GBD, producing comparable and consistent estimates of PAH burden. We used epidemiological and vital registry data to estimate the non-fatal and fatal burden of PAH in 204 countries and territories from 1990 to 2021 using standard GBD modelling approaches. We specifically focused on PAH (group 1 pulmonary hypertension), and did not include pulmonary hypertension groups 2–5.

**Findings:**

In 2021, there were an estimated 192 000 (95% uncertainty interval [UI] 155 000–236 000) prevalent cases of PAH globally. Of these, 119 000 (95 900–146 000) were in females (62%) and 73 100 (58 900–89 600) in males (38%). The age-standardised prevalence was 2·28 cases per 100 000 population (95% UI 1·85–2·80). Prevalence increased with age such that the highest prevalence was among individuals aged 75–79 years. In 2021, there were 22 000 deaths (18 200–25 400) attributed to PAH globally, with an age-standardised mortality rate of 0·27 deaths from PAH per 100 000 population (0·23–0·32). The burden of disease appears to be improving over time (38·2% improvement in age-standardised years of life lost [YLLs] in 2021 relative to 1990). YLLs attributed to PAH were similar to estimates for conditions such as chronic myeloid leukaemia, multiple sclerosis, and Crohn's disease.

**Interpretation:**

PAH is a rare but fatal disease that accounts for a considerable health-associated burden worldwide. PAH is disproportionally diagnosed among females and older adults.

**Funding:**

Cardiovascular Medical Research and Education Fund and the Bill & Melinda Gates Foundation.

## Introduction

Pulmonary arterial hypertension (PAH) is a rare disease leading to progressive remodelling and narrowing of the pulmonary vasculature, right heart failure, and death.^1^, ^2^ Although targeted therapies for PAH have been developed and contribute to improved morbidity and mortality, persistent disability and continued high health-care costs present ongoing challenges for patients and health-care systems.^3^, ^4^, ^5^ Risk factors for PAH (such as HIV, connective tissue disease, and methamphetamine use), access to resource-intensive subspecialty care, and outcomes are thought to vary worldwide, but comprehensive global estimates of PAH burden have not been previously published.^6^, ^7^, ^8^, ^9^, ^10^, ^11^, ^12^ There is a need to better estimate the global and regional burden of PAH, including the use of more comprehensive estimates of burden such as years of life lost (YLLs) or disability-adjusted life-years (DALYs) to better inform health policy and health system decision makers.

The Global Burden of Diseases, Injuries, and Risk Factors Study (GBD) is an ongoing, multinational research project that quantifies health loss across the world. GBD collects all available data and applies statistical and epidemiological models to produce comparable, consistent, and comprehensive estimates of disease burden across age, sex, geographical location, and year. The study will now include PAH as a cause for the first time, producing summary estimates of YLLs, years lived with disability (YLDs), DALYs, and traditional measures of non-fatal and fatal burden.

The aim of this investigation was to better characterise the worldwide epidemiology of PAH including regional variation, place this in context relative to other disease entities, and highlight areas of uncertainty that necessitate further study. This effort was specifically focused on PAH (group 1 pulmonary hypertension). By adding PAH to the list of conditions in GBD, this initial report will be revisited over time to reflect new data and changes in population health around the world.

**Research in context**

**Evidence before this study**

We searched the Global Index Medicus and PubMed for keywords related to pulmonary arterial hypertension (PAH) between 1980 and 2021 to identify population-representative sources of prevalence, incidence, and mortality with clinically diagnosed PAH (additional details in appendix 1 pp 21–23). Of 6772 articles identified, we found 65 with population-level data, the majority of which were focused on mortality. Previous studies looked mainly at single geographies or limited subsets of disease such as idiopathic PAH, global estimates of PAH were extrapolated, and there was little to no assessment of disease burden outside of incidence, prevalence, and mortality.

**Added value of this study**

This study extends previous evidence by establishing a global estimate for the non-fatal and fatal burden of PAH, which is a disease that is recognised to cause lifelong disability. PAH disability was measured by prevalence, mortality, and disability estimates (eg, disability-adjusted life-years and years of life lost), which suggested a burden of disease similar to that of more common but less fatal diseases. PAH is more commonly diagnosed in females, has a similar burden between males and females, and disproportionately affects older adults. Estimates of mortality appear to be improving over time. PAH remains a difficult disease to diagnose clinically, case definitions have changed over time, and data in many parts of the world remain sparse. These challenges likely impact these estimates and might blunt the recognition of more substantial regional variation.

**Implications of all the available evidence**

PAH has not historically been a strong focus of global public health discussion and there are certainly diseases with a larger public health burden. Nevertheless, there are an increasing number of effective therapies for PAH and the development of PAH has been linked with relatively common exposures such as schistosomiasis and methamphetamine use. These factors highlight the importance of developing tools to simplify the diagnosis and strengthen public health reporting systems to monitor this disease to more effectively target resources and research in the future. In addition, future research should focus on the contributions to global burden that are made by modifiable risk factors.

## Methods

### Overview

GBD estimates disease burden for 371 diseases and injuries and 88 risk factors in 204 countries and territories. Further information on the location, disease, and risk hierarchies used for GBD 2021 can be found on the Global Health Data Exchange (GHDx). In 2021, PAH was added as its own condition in GBD, producing comparable and consistent estimates of PAH burden from 1990 to 2021. The current haemodynamic definition of PAH includes a resting mean pulmonary arterial pressure of more than 20 mm Hg, pulmonary capillary wedge pressure of less than 15 mm Hg, and pulmonary vascular resistance of at least 2 Wood units obtained by right heart catheterisation; however, this haemodynamic definition has changed over time and is similar but not identical to definitions that would have been used by physicians over the study period.^2^, ^13^ Echocardiography is also used to estimate pulmonary arterial pressure, a practice that is common in resource-limited settings and can influence the sensitivity and specificity of the diagnosis.

A standard case definition was used by GBD when identifying data sources to include. The GBD case definition required a physician diagnosis of PAH in which there was supporting evidence for the diagnosis from either right heart catheterisation or echocardiography. Independent review of haemodynamics or echocardiography was not performed; we merely required an assurance that corroborating evidence from either of these modalities informed physician diagnoses used in the included data source. We included PAH identified by ICD codes if the diagnosis was confirmed by reviewing the medical records. Pulmonary hypertension can arise from several different cardiopulmonary diseases as designated by classification groups. The current effort was focused on PAH (group 1 pulmonary hypertension). PAH is distinct from pulmonary hypertension more broadly, which can arise from a variety of other heart and lung diseases. Pulmonary hypertension groups 2–5 have distinct pathophysiology, treatments, and natural history and were not included.^2^

GBD uses de-identified data, and the waiver of informed consent was reviewed and approved by the University of Washington Institutional Review Board (study number 9060). This manuscript was produced as part of the GBD Collaborator Network and in accordance with the GBD Protocol. This study complies with the Guidelines for Accurate and Transparent Health Estimates Reporting (GATHER); a checklist can be found in appendix 1 (p 3).^14^

### Mortality estimation

The Cause of Death Ensemble model (CODEm) software was used to estimate mortality due to PAH.^15^ A brief description follows; full details, including covariates and model settings, can be found in appendix 1 (pp 6–12). CODEm generates a set of plausible submodels using multiple statistical families and combinations of covariates. Possible covariates were identified based on a priori understanding of the association between the covariate and PAH. Combinations of covariates were tested for statistical significance and plausibility (eg, coefficients must have the expected directions based on information about disease pathophysiology in the literature, inputs from disease experts, and a priori expectations about the relationships between access to health care or development level of a population and mortality). The final ensemble model was a weighted average of the available submodels, where the weights were determined by submodel performance in out-of-sample predictive validity testing.

Vital registration records were used as input data for estimating PAH mortality in CODEm. Vital registration records were mapped to the GBD cause hierarchy by ICD code. ICD codes 416.0 (ICD-9) and I27.0 (ICD-10) were used as both are specific to PAH. A revision of the ICD-10 coding system revealed an issue with deaths coded to I27.0 for some years and locations. This revision introduced the use of I27.2 to code for deaths due to other types of pulmonary hypertension. In some countries, the introduction of I27.2 caused large changes in the patterns of deaths coded to I27.0, either in level or temporal trend. As we do not expect large changes in PAH-related mortality, the most likely explanation was that before the regular use of I27.2, non-PAH types of pulmonary hypertension were often erroneously coded to I27.0. We thus concluded that the most accurate and conservative estimates of PAH burden should be based on deaths coded to I27.0 after the introduction of I27.2 and excluded all input data for affected locations before the introduction of I27.2 (additional details included in appendix 1 pp 18–19). In addition, non-specific, intermediate, or implausible causes of death in vital registration records were reassigned to valid underlying causes of death (including PAH) via a set of redistribution algorithms that use (1) proportional information, (2) cause-specific priors, or (3) datasets with complete information on all contributing causes of death in addition to the underlying cause.

### Morbidity estimation

We performed a systematic review using the Global Index Medicus and PubMed databases to gather epidemiological studies reporting data for PAH from 1980 to 2021. Complete details of this review have been previously published.^11^ Search terms, information on inclusion and exclusion criteria including a PRISMA diagram, and resultant data are described in appendix 1 (pp 20–25). Data sources included in the model can be accessed via GHDx, a publicly available, searchable database of model inputs. Full details of all data processing steps and model settings can be found in appendix 1 (pp 20–25).

We used Disease Modeling Meta-Regression (DisMod-MR) version 2.1 to estimate non-fatal burden of PAH by age group, sex, location, and year.^16^ DisMod-MR is a Bayesian modelling meta-regression tool that uses disease parameters (eg, incidence, prevalence, remission), epidemiological relationships between these parameters, predictive covariates, and a geographical hierarchy (global, super-region, region, country, subnational) to generate internally consistent estimates of non-fatal burden. Using priors generated at each level of the geographical hierarchy and coefficients for included covariates, DisMod-MR generates predictions for all locations, including those without epidemiological input data. Additional description of these methods is included in appendix 1 (pp 13–17).

Estimates reported in epidemiological studies often lack demographic detail. We split both-sex data points into male and female estimates using the tool MR-BRT (Meta-Regression, Bayesian, Regularized, Trimming).^17^ To do this, we modelled the log-ratio of male to female values, and then used location-specific population structures to calculate sex-specific estimates. We split all-age data points into bins with a 10-year age range. To do this, we estimated the global age-pattern of PAH with a DisMod-MR model using only data where the span between the minimum age value and maximum age value for a data point was less than 25 years. The results from this model and information on location-specific population structures were used to calculate the split values.

### Summary measure methods

GBD produces summary measures of disease burden, including YLLs, YLDs, and DALYs. YLLs were calculated as the difference between age at death and an ideal life expectancy calculated using a reference life table to identify the highest observed life expectancy for each age.^15^ YLDs were calculated as the product of prevalent cases and disability weights to capture differences in burden by disease state.^16^ As PAH results in right heart failure, the disability weights specific to heart failure were selected to represent the disability due to PAH. DALYs were calculated as the sum of YLDs and YLLs.

### General statistical methods

Estimates were generated for each GBD age group, location, year, and sex. Uncertainty was estimated by sampling from the posterior distributions at each step in the modelling process and is reported as 95% uncertainty intervals (UIs), with lower and upper bounds calculated as the 2·5% and 97·5% quantile. Three significant figures were used when reporting estimates. While this is consistent with convention in GBD, this means that component estimates presented in the manuscript (eg, males and females) cannot always be added to generate the reported total. We used the direct method with the GBD standard global population to produce age-standardised estimates. The GBD standard population is based on the population structure of all countries with populations greater than 5 million people. In a two-step process, the proportion of the location-specific population in each age group is calculated and then these age-specific proportions are averaged across all locations.^15^ Code used for GBD estimation is publicly available online.

### Role of the funding source

The funders of the study had no role in study design, data collection, data analysis, data interpretation, or writing of the report.

## Results

**Figure 1:**
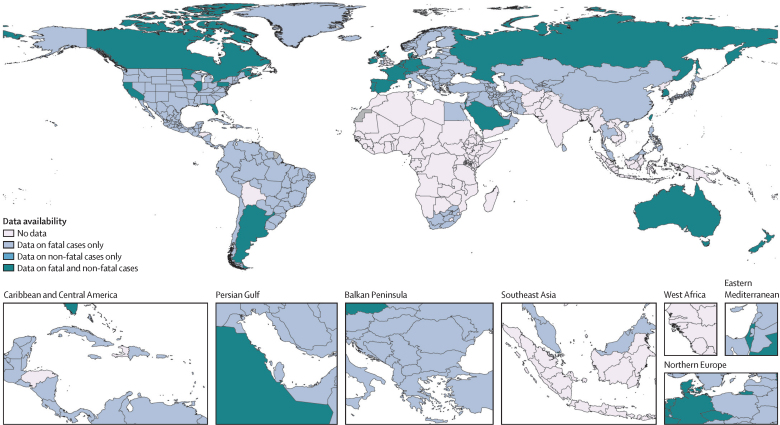
Availability of data to inform global burden of disease models for pulmonary arterial hypertension These estimates are based on data from vital registration (births, deaths, and cause of death), non-vital registration sources (population registries, census, and other sources), or both.

Consistent with previous GBD analyses, source data that were used to inform the appropriate models to estimate fatal and non-fatal metrics for PAH were broadly but not universally available (figure 1). Additional details about studies used to inform morbidity estimates, including locations, years, and methods used to confirm the diagnosis of PAH, are included in appendix 1 (pp 20–25).

**Figure 2:**
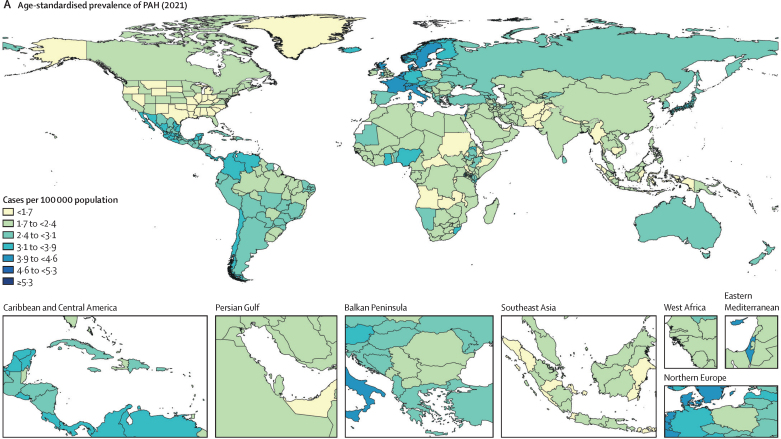

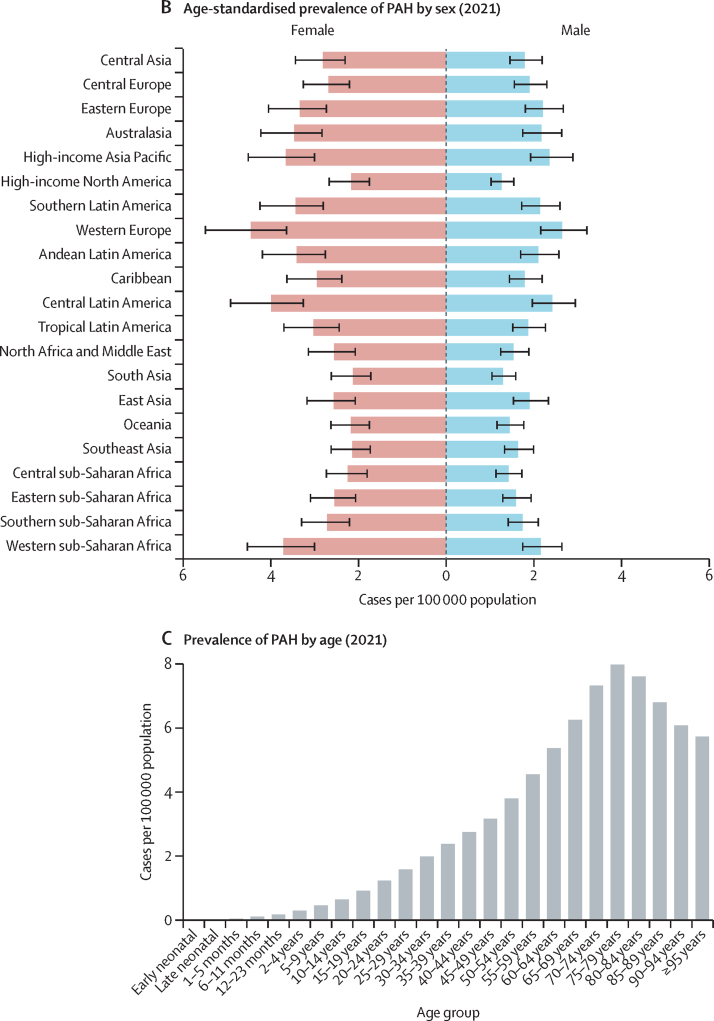
PAH prevalence (A) Age-standardised prevalence of PAH by region in 2021 (both sexes). (B) Age-standardised prevalence of PAH by sex and region in 2021; error bars show 95% uncertainty intervals. (C) Global age-specific prevalence of PAH in 2021. PAH=pulmonary arterial hypertension.

In 2021, there were 192 000 prevalent cases (95% UI 155 000–236 000) of PAH. The age-standardised prevalence of PAH was 2·28 cases per 100 000 population (95% UI 1·85–2·80). Estimates of PAH prevalence appeared to be relatively stable over time, with an age-standardised prevalence of PAH in 1990 of 2·30 per 100 000 population (1·87–2·82; table 1). PAH was more prevalent among females, with 2·75 cases of PAH per 100 000 females (2·24–3·39) versus 1·78 cases of PAH per 100 000 males (1·44–2·17) in 2021. Of the 192 000 individuals with prevalent PAH, 119 000 (95 900–146 000) were female (62%) and 73 100 (58 900–89 600) were male (38%). Figure 2 shows the age-standardised prevalence and distribution of PAH by sex and age globally. There was a similar PAH prevalence worldwide, albeit with slightly higher prevalence in western Europe, central Latin America, and high-income Asia Pacific, and a lower prevalence in south Asia, high-income North America, and Oceania. Age-standardised prevalence by region ranged from 1·71 cases per 100 000 population (1·38–2·09) in south Asia to 3·56 cases per 100 000 population (2·92–4·35) in western Europe. Prevalence of PAH increased with age, with the highest prevalence observed in individuals aged 75–79 years (7·99 per 100 000, 5·32–11·6). All-age counts of prevalence by region and sex are included in appendix 1 (p 5).

**Table 1:** Global age-standardised prevalence of pulmonary arterial hypertension per 100 000 population and age-standardised mortality attributed to pulmonary arterial hypertension per 100 000 population from 1990 to 2021

	**Prevalence**			**Mortality**		
	Male	Female	Total	Male	Female	Total
1990	1·75 (1·42–2·13)	2·81 (2·29–3·46)	2·30 (1·87–2·82)	0·37 (0·29–0·46)	0·34 (0·23–0·45)	0·35 (0·29–0·42)
1995	1·75 (1·42–2·12)	2·81 (2·29–3·46)	2·29 (1·86–2·82)	0·36 (0·29–0·45)	0·33 (0·25–0·46)	0·35 (0·29–0·41)
2000	1·75 (1·42–2·13)	2·78 (2·26–3·42)	2·28 (1·85–2·80)	0·33 (0·28–0·41)	0·33 (0·26–0·44)	0·33 (0·29–0·39)
2005	1·79 (1·45–2·18)	2·81 (2·29–3·46)	2·31 (1·88–2·84)	0·32 (0·27–0·38)	0·32 (0·26–0·42)	0·32 (0·28–0·37)
2010	1·80 (1·46–2·19)	2·81 (2·29–3·44)	2·32 (1·88–2·83)	0·35 (0·27–0·41)	0·34 (0·26–0·41)	0·34 (0·28–0·39)
2015	1·82 (1·47–2·21)	2·81 (2·28–3·45)	2·32 (1·88–2·84)	0·32 (0·24–0·38)	0·31 (0·25–0·38)	0·32 (0·26–0·36)
2020	1·80 (1·46–2·19)	2·77 (2·25–3·41)	2·29 (1·86–2·81)	0·27 (0·21–0·33)	0·28 (0·22–0·35)	0·28 (0·23–0·32)
2021	1·78 (1·44–2·17)	2·75 (2·24–3·39)	2·28 (1·85–2·80)	0·27 (0·21–0·33)	0·28 (0·22–0·34)	0·27 (0·23–0·32)

Data are number of cases or number of deaths per 100 000 population, with 95% uncertainty intervals in parentheses.

**Figure 3:**
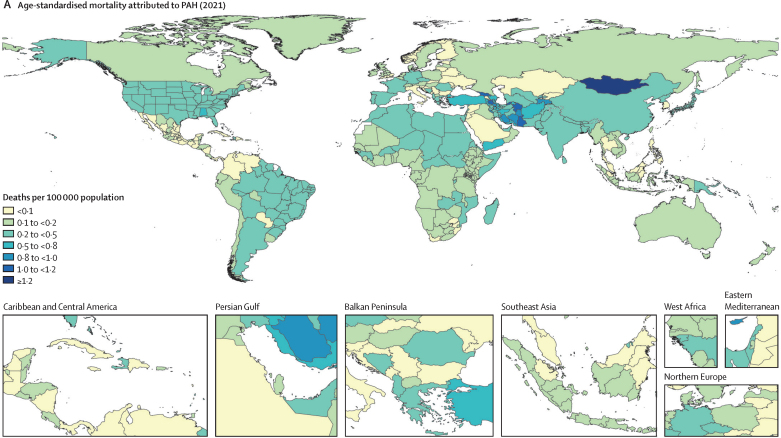

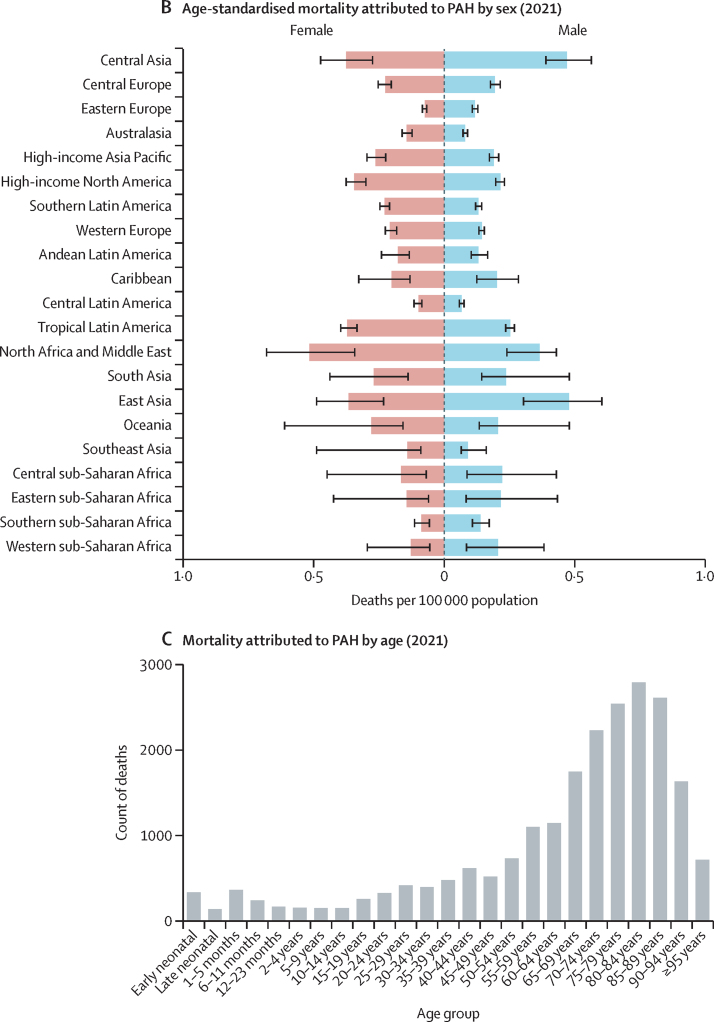
PAH mortality (A) Age-standardised mortality attributed to PAH by region in 2021 (both sexes). (B) Age-standardised mortality attributed to PAH by sex and region in 2021; error bars show 95% uncertainty intervals. (C) Total global deaths from PAH by age group in 2021. PAH=pulmonary arterial hypertension.

In 2021, there were 22 000 deaths (95% UI 18 200–25 400) attributed to PAH globally. The age-standardised mortality rate attributed to PAH (cause-specific mortality) in 2021 was 0·27 deaths per 100 000 population (95% UI 0·23–0·32). Cause-specific mortality improved slowly over time as the 2021 estimate was lower than the estimated rate in 1990 of 0·35 deaths from PAH per 100 000 population (0·29–0·42), a decrease of 17·6% (table 1). Despite a higher prevalence of PAH in females, cause-specific mortality in the population was similar between females and males, with 0·28 deaths from PAH per 100 000 females (0·22–0·34) and 0·27 deaths from PAH per 100 000 males (0·21–0·33) in 2021. Figure 3 shows cause-specific mortality per 100 000 population and the distribution by sex and age globally. Mortality attributed to PAH was similar worldwide, albeit with slightly higher cause-specific mortality in north Africa and the Middle East, central Asia, and east Asia, and lower mortality in central Latin America, eastern Europe, and southern sub-Saharan Africa. Age-standardised cause-specific mortality by region ranged from 0·08 deaths from PAH per 100 000 population (0·07–0·10) in central Latin America to 0·44 deaths from PAH per 100 000 population (0·31–0·53) in north Africa and the Middle East. All-age counts of mortality by region and sex are included in appendix 1 (p 5).

The health-related burden of PAH is shown in table 2. PAH contributed 642 000 DALYs (53·3% among females and 46·7% among males) in 2021. The strongest driver of burden was in YLLs; PAH accounted for 624 000 YLLs (53·3% among females and 46·6% among males) in 2021. The burden appeared to be gradually improving over time, with a 37·6% improvement in age-standardised DALYs per 100 000 people and a 38·2% improvement in age-standardised YLLs per 100 000 people when compared with 1990 (appendix 1 p 4). To contextualise the burden of PAH from the vantage of DALYs and YLLs relative to other conditions evaluated by GBD, a selection of conditions with YLLs within approximately ±10 YLLs per 100 000 population are presented in table 3. PAH had a burden of YLLs similar to that of thyroid disease, chronic myeloid leukaemia, testicular cancer, and Crohn's disease.

**Table 2:** Absolute and relative global burden of pulmonary arterial hypertension in 2021, including DALYs, YLDs, and YLLs

	**Total burden across all ages**	**Age-standardised rate per 100 000 population**
**DALYs**		
Female	342 000 (283 000–431 000)	8·39 (6·92–10·5)
Male	300 000 (247 000–348 000)	8·06 (6·72–9·36)
Total	642 000 (552 000–729 000)	8·24 (7·14–9·39)
**YLDs**		
Female	11 000 (7220–16 200)	0·25 (0·17–0·38)
Male	6770 (4420–9910)	0·17 (0·11–0·24)
Total	17 800 (11 700–25 900)	0·21 (0·14–0·31)
**YLLs**		
Female	331 000 (273 000–420 000)	8·14 (6·66–10·3)
Male	293 000 (240 000–343 000)	7·9 (6·55–9·23)
Total	624 000 (536 000–715 000)	8·03 (6·95–9·21)

Data in parentheses are 95% uncertainty intervals. DALYs=disability-adjusted life-years. YLDs=years lived with disability. YLLs=years of life lost.

**Table 3:** Global age-standardised YLLs and DALYs of other representative conditions relative to pulmonary arterial hypertension in 2021

	**YLLs per 100 000 population**	**DALYs per 100 000 population**
Malignant melanoma	18·1 (15·7–19·7)	19·6 (17·3–21·5)
Thyroid diseases	9·62 (8·29–10·9)	89·7 (56·9–137)
Chronic myeloid leukaemia	8·17 (5·76–10·5)	8·3 (5·86–10·6)
Pulmonary arterial hypertension	8·03 (6·95–9·21)	8·24 (7·14–9·39)
Testicular cancer	6·36 (6·01–6·73)	6·91 (6·51–7·36)
Multiple sclerosis	5·68 (5·41–5·98)	11·4 (9·77–13·2)
Crohn's disease	2·00 (1·76–2·29)	4·65 (3·69–5·85)

Data in parentheses are 95% uncertainty intervals. DALYs=disability-adjusted life-years. YLLs=years of life lost.

Further country-specific, cause-specific, non-fatal, and fatal estimates, including the comparison with other disease states, can be found on GBD Compare.

## Discussion

Despite its relative rarity, PAH imposes a considerable health burden worldwide. In 2021, there were 2·28 prevalent cases of PAH per 100 000 population, with a mortality rate of 0·27 deaths from PAH per 100 000 population. According to our analyses, the burden of PAH as measured by YLLs was similar to that seen in conditions such as chronic myeloid leukaemia, and higher than that seen in conditions such as multiple sclerosis, Crohn's disease, and testicular cancer. There was modest variability across a relatively narrow range in both the prevalence and cause-specific mortality from PAH worldwide. The burden from PAH might be modestly improving over time.

Previous studies, largely anchored to local or national registries, have reported a wide range for PAH prevalence, from 0·37 cases per 100 000 in a French paediatric registry to 15 cases per 100 000 in an Australian echocardiography database.^18^, ^19^ The current GBD prevalence estimate of 2·28 prevalent cases of PAH per 100 000 population using a more standardised and comprehensive dataset is similar to the mean of prevalence estimates in these previously reported studies, but substantially higher than estimates reported in previous reviews of global PAH prevalence that relied on fewer data sources.^7^, ^11^

Regional variations were observed in estimates of prevalence and mortality. In regions with high prevalence, the difference was approximately 2-fold higher compared with regions with low prevalence. Similarly, regions with high cause-specific mortality had an approximately 5-fold higher rate than those with lower mortality. These regional differences might reflect an uneven regional distribution of risk factors implicated in the pathogenesis of PAH. For example, genetic predisposition, methamphetamine use, supplement use, and infections such as schistosomiasis or HIV (among a number of potential risk factors) vary by region and might drive local differences in PAH prevalence.^20^, ^21^, ^22^ In addition, competing risks from infection, trauma, and other diseases are uneven globally and could impact the population at risk of developing PAH.^23^ It is also possible that some of the observed variation reflects regional differences in what is recognised as PAH and reporting systems in place. This concern might be magnified in regions where access to diagnostic services is limited.

Regions with the highest prevalence of PAH were not always regions with the highest cause-specific PAH mortality. This finding could reflect regional differences in case fatality rates driven by the availability of treatment or differential comorbidities that have a substantial impact on outcomes. Alternatively, in a recurrent theme, some of the difference might be explained by regional differences in disease attribution. Disease labelled as PAH might be distinct in different regions and might include entities with a higher or lower case fatality rate in different regions.^24^ PAH is diagnostically complex and there is ample evidence that the diagnostic formulation can shift between PAH and non-PAH pulmonary hypertension as patients move between health-care teams.^2^, ^25^, ^26^, ^27^ Although not specifically reported for PAH, differential classification of disease by region is relatively common and it would not be surprising if the diagnostic formulation was influenced by the local practice environment.^24^, ^28^ Fortunately, while non-trivial relative differences were noted, absolute differences in regional prevalence and cause-specific mortality were small as a fraction of the overall population. This observation might suggest that the effect of these combined factors is similarly small on a population basis.

The current estimates support the long-standing recognition that PAH is more common in females but refute the historical observation that PAH is predominantly a diagnosis of youth.^29^, ^30^, ^31^ The observation that PAH is increasingly a diagnosis among older adults has been noted in recent registry-based reports and is reflected in the GBD estimates.^7^, ^32^, ^33^, ^34^, ^35^ PAH prevalence was highest among individuals aged 75–79 years in the GBD estimates. Previous studies have suggested that PAH among older adults lacks the same predilection towards females, has different clinical features, and lacks a robust response to medical therapy.^33^ This raises questions about whether the increasingly older distribution of PAH represents monolithic pulmonary vascular disease, a separate novel entity, misattributed pulmonary hypertension from heart or lung disease, or a combination of these factors. GBD is not well positioned to answer these questions; however, the current estimates can confirm that physicians are increasingly labelling cardiopulmonary diseases as PAH among older adults worldwide, and further research is needed to understand the key elements driving this shift in population-level demographics.

The current study offers comprehensive cause-specific mortality rates and is the first to provide global estimates for the health-related burden associated with PAH, including DALYs, YLDs, and YLLs. The burden associated with PAH was substantial, with a global impact of 642 000 DALYs contributed by PAH. Despite a higher prevalence in females, the burden of disease was similar for males and females, which supports the hypothesis that the disease course might be more aggressive in males. The sex paradox in PAH—higher prevalence in females but higher case fatality rate in males—is well recognised but not fully explained, and could be related to sex hormones, health behaviours, and differences in right heart adaptation among other factors.^29^ Not surprisingly for a progressive and often fatal disease, the major contribution to health-related burden was a result of YLLs. The YLLs attributable to PAH were similar to more common but less fatal diseases such as multiple sclerosis and chronic myeloid leukaemia. Nevertheless, estimates of burden appear to be improving over time. Our work does not fully clarify whether these improvements are related to improved treatment, changing disease definitions, risk factor mitigation, or competing risks driving mortality towards other causes.

This work establishes a baseline for ongoing longitudinal monitoring of PAH prevalence against which future deviations can be compared or against which a health-care system can estimate the degree to which PAH diagnoses might be missed. For example, the recognition that anorexigens or methamphetamine caused PAH took time, which delayed efforts to make proactive changes to prevent PAH in at-risk populations.^2^, ^20^, ^36^ Estimation of PAH burden in GBD could be an important epidemiological tool to identify emerging problems contributing to higher local or regional prevalence. This recognition, in turn, could focus research and resources to better prevent, identify, and treat PAH in the future.

Importantly, we present estimates of recognised PAH. A substantial burden of PAH might be unrecognised, and the case definition used for this study might not sensitively identify relevant subtypes of PAH.^37^ As a result of these factors, the burden of PAH could be higher than we estimate, and unrecognised or uncounted disease might contribute to heterogeneity by country if resource constraints limited diagnosis. Relatedly, although reliable subtyping of PAH by aetiology is of interest to the field, it was not possible in GBD at this time. Further limitations include data sparsity in many regions, evolving disease definitions for PAH, shifting approaches to diagnosis, and evolving coding practices, including the transition between ICD-9 and ICD-10 coding. Coding practices have evolved over time, with no specific codes for PAH until the early 2000s. We performed an analysis of primary mortality data, chose to remove country-years where I27.0 was affected, and excluded I27.2. This biases our earlier estimates towards the present-day average because we had fewer data to inform our estimates in earlier years and might underestimate the number of deaths attributable to PAH, particularly for some subtypes of disease more heavily coded using I27.2 in some regions. Similarly, data sparsity in resource-poor countries and extrapolation from other regions could bias estimates towards those seen in more well resourced countries. This could blunt meaningful regional differences. Finally, as discussed, the clinical diagnosis of PAH is complex and misclassification or differential classification might be common, such that the diagnosis could be missed or PAH estimates could be inflated by pulmonary hypertension from other causes; however, while there is always room to improve the number and source of inputs, the relatively narrow range of absolute regional variation is reassuring and suggests that differences in attribution by country are not a major source of bias at a population level.

While data limitations are noted and estimates might be considered a lower limit of the burden of PAH, the relatively high level of data availability and consistency across regions is notable for a disease that until recently was considered an orphan disease. Moving forward, ongoing attention to straightforward disease definitions, advances in testing and screening, and stronger reporting systems remain vitally important to ensure that countries are consistently capturing the same disease entity when PAH is diagnosed and approximating the full burden from this condition. These health policy foci will be increasingly important as costly treatments for PAH continue to be developed, the population continues to age, and emerging risk factors such as the methamphetamine epidemic continue to spread. This vigilance will ensure that resources can be allocated appropriately, local risk factors are mitigated, drugs are sustainably targeted, and health systems are not systematically missing PAH diagnoses.

In conclusion, by leveraging all available data alongside covariates and models, this study reveals global PAH epidemiology at a greater demographic granularity than previously published. We estimate that at least 192 000 people had PAH in 2021, that 22 000 died from the disease, and that PAH contributed to 624 000 YLLs globally. PAH is more common among females and older adults. Greater understanding of global epidemiology can direct resources and research towards locations, sexes, and age groups most in need, and could identify emerging exposures that might cause PAH in the future.

### GBD 2021 Pulmonary Arterial Hypertension Collaborators

### Contributors

### Data sharing

Our study follows the Guidelines for Accurate and Transparent Health Estimates Reporting (GATHER; appendix 1 p 3). The findings of this study are supported by data available in public online repositories, data publicly available upon request of the data provider, and data not publicly available due to restrictions by the data provider. Non-publicly available data were used under license for the current study but may be available from the authors upon reasonable request and with permission of the data provider. Details of data sources and availability can be found online in the GBD Sources Tool (https://ghdx.healthdata.org/gbd-2021/sources). The full output of the analyses is publicly available in the Global Health Data Exchange (https://vizhub.healthdata.org/gbd-results/) and can further be explored via customised data visualisation tools (https://vizhub.healthdata.org/gbd-compare/).

## Declaration of interests

O C Baltatu reports support for the present manuscript from the National Council for Scientific and Technological Development (CNPq, 304224/2022-7) and the Anima Institute through an AI research professor fellowship; and a leadership or fiduciary role with the Health and Biotechnology Advisory Board at Technology Park São José dos Campos – Center for Innovation in Health Technologies (CITS) outside the submitted work. S Bhaskar reports grants or contracts from the Japan Society for the Promotion of Science (JSPS), Japanese Ministry of Education, Culture, Sports, Science and Technology (MEXT) through a Grant-in-Aid for Scientific Research (KAKENHI), and from JSPS and the Australian Academy of Science through a JSPS International Fellowship; and leadership or fiduciary role in other board, society, committee or advocacy group, paid or unpaid, with Rotary District 9675, the Global Health & Migration Hub Community, Global Health Hub Germany, Berlin, with *PLOS One, BMC Neurology, Frontiers in Neurology, Frontiers in Stroke, Frontiers in Public Health*, and *BMC Medical Research Methodology* as an Editorial Board Member, and as a member of the College of Reviewers, Canadian Institutes of Health Research (CIHR), Government of Canada; all outside the submitted work. P A Corris reports participation on a data safety monitoring board or advisory board with Aerovate and Merck; and leadership or fiduciary role in other board, society, committee, or advocacy group, paid or unpaid, as Chairman of the Pulmonary Vascular Research Institute; all outside the submitted work. H M DuBrock reports a research grant from Bayer Pharmaceuticals; consulting fees from Merck; and participation on an advisory board with Merck and Janssen Pharmaceuticals; all outside the submitted work. M Hultström reports support for the present manuscript from the Swedish Heart-Lung Foundation and Knut och Alice Wallenberg Foundation through payments to their institution; grants or contracts from the Swedish Society of Medicine through their institution; payment or honoraria for lectures, presentations, speakers bureaus, manuscript writing or educational events from the Swedish Society for Anaesthesiology and Intensive Care Medicine; support for attending meetings or travel from the American Physiological Society; and leadership or fiduciary role in other board, society, committee, or advocacy group, paid or unpaid, with the American Physiological Society as a Steering Committee Member of the section for Water and Electrolyte Homeostasis; all outside the submitted work. N E Ismail reports a leadership or fiduciary role in other board, society, committee or advocacy group, unpaid, with the Malaysian Academy of Pharmacy, Malaysia, and the Malaysian Pharmacists Society Education Chapter outside the submitted work. J J Jozwiak reports payment or honoraria for lectures, presentations, speakers bureaus, manuscript writing, or educational events from Novartis, Adamed, and Amgen, outside the submitted work. K Krishan reports non-financial support from the UGC Centre of Advanced Study, CAS II, awarded to the Department of Anthropology, Panjab University, Chandigarh, India, outside the submitted work. P J Leary reports grants or contracts from the National Heart, Lung, and Blood Institute through payments to them and their institution (R33/R61: Famotidine RCT; R01: Multi-omics; R33/R61: Valsartan; R01: Methamphetamine PAH), from Bayer through payments to their institution (Mentor on a mentored award about FDG-uptake in PAH; no honoraria or salary support), from the Cystic Fibrosis Foundation Therapeutic Development Network through payments to them and their institution, and from Janssen Pharmaceuticals through payments to a third party for data acquisition and analysis; consulting fees from Sumitomo Pharma as personal payments; participation on a data safety monitoring board with the National Heart, Lung, and Blood Institute; leadership or fiduciary role, unpaid, with the Pulmonary Hypertension Association Scientific Leadership Council and the Team Phenomenal Hope Steering Committee; and receipt of medical writing support and collation of co-author feedback on a big-data locations of care project (under review) from Janssen Pharmaceuticals; all outside the submitted work. M-C Li reports grants or contacts from the National Science and Technology Council, Taiwan (NSTC 113-2314-B-003-002); and leadership or fiduciary role in other board, society, committee or advocacy group, paid or unpaid, with the *Journal of the American Heart Association*; all outside the submitted work. J A Singh reports consulting fees from ROMTech, Atheneum, ClearView Healthcare Partners, American College of Rheumatology, Yale, Hulio, Horizon Pharmaceuticals, DINORA, Frictionless Solutions, Schipher, Crealta/Horizon, Medisys, Fidia, PK Med, Two Labs Inc, Adept Field Solutions, Clinical Care Options, Putnam Associates, Focus Forward, Navigant Consulting, Spherix, MedIQ, Jupiter Life Science, UBM LLC, Trio Health, Medscape, WebMD, and Practice Point Communications, and the National Institutes of Health; payment or honoraria for lectures, presentations, speakers bureaus, manuscript writing, or educational events on the speakers bureau of Simply Speaking; support for attending meetings or travel from OMERACT as a steering committee member; participation on a data safety monitoring board or advisory board with the FDA Arthritis Advisory Committee; leadership or fiduciary role in other board, society, committee, or advocacy group, paid as a past steering committee member of the OMERACT, an international organisation that develops measures for clinical trials and receives arm's length funding from 12 pharmaceutical companies, unpaid as Chair of the Veterans Affairs Rheumatology Field Advisory Committee, and unpaid as the Editor and Director of the UAB Cochrane Musculoskeletal Group Satellite Center on Network Meta-analysis; stock or stock options in Atai life sciences, Kintara Therapeutics, Intelligent Biosolutions, Acumen Pharmaceutical, TPT Global Tech, Vaxart Pharmaceuticals, Atyu Biopharma, Adaptimmune Therapeutics, GeoVax Labs, Pieris Pharmaceuticals, Enzolytics Inc, Seres Therapeutics, Tonix Pharmaceuticals Holding Corp, Aebona Pharmaceuticals, and Charlotte's Web Holdings, Inc; and previous stock options in Amarin, Viking, and Moderna Pharmaceuticals; all outside the submitted work. C E Ventetuolo reports grants or contracts from the National Institutes of Health and American Heart Association through payments to their institution; consulting fees from Merck, Janssen, United Therapeutics, and Regeneron; payment or honoraria for lectures, presentations, speakers bureaus, manuscript writing, or educational events from Dynamed; and support for attending meetings or travel from the American Thoracic Society; all outside the submitted work. M Zielińska reports other financial or non-financial interests in AstraZeneca as an employee, outside the submitted work. All other authors declare no competing interests.

## References

[1] Dresdale DT, Schultz M, Michtom RJ (1951). Primary pulmonary hypertension. I. Clinical and hemodynamic study. Am J Med.

[2] Humbert M, Kovacs G, Hoeper MM (2023). 2022 ESC/ERS Guidelines for the diagnosis and treatment of pulmonary hypertension. Eur Respir J.

[3] McLaughlin VV, Shah SJ, Souza R, Humbert M (2015). Management of pulmonary arterial hypertension. J Am Coll Cardiol.

[4] Gu S, Hu H, Dong H (2016). Systematic review of the economic burden of pulmonary arterial hypertension. PharmacoEconomics.

[5] Noel ZR, Kido K, Macaulay TE (2017). Selexipag for the treatment of pulmonary arterial hypertension. Am J Health Syst Pharm.

[6] Hasan B, Hansmann G, Budts W (2020). Challenges and special aspects of pulmonary hypertension in middle- to low-income regions: JACC state-of-the-art review. J Am Coll Cardiol.

[7] Hoeper MM, Humbert M, Souza R (2016). A global view of pulmonary hypertension. Lancet Respir Med.

[8] Harikrishnan S, Sanjay G, Ashishkumar M (2018). Pulmonary hypertension registry of Kerala, India (PRO-KERALA)—clinical characteristics and practice patterns. Int J Cardiol.

[9] Thienemann F, Dzudie A, Mocumbi AO (2016). The causes, treatment, and outcome of pulmonary hypertension in Africa: insights from the Pan African Pulmonary Hypertension Cohort (PAPUCO) Registry. Int J Cardiol.

[10] Rich S, Haworth SG, Hassoun PM, Yacoub MH (2018). Pulmonary hypertension: the unaddressed global health burden. Lancet Respir Med.

[11] Emmons-Bell S, Johnson C, Boon-Dooley A (2022). Prevalence, incidence, and survival of pulmonary arterial hypertension: a systematic review for the global burden of disease 2020 study. Pulm Circ.

[12] Leber L, Beaudet A, Muller A (2021). Epidemiology of pulmonary arterial hypertension and chronic thromboembolic pulmonary hypertension: identification of the most accurate estimates from a systematic literature review. Pulm Circ.

[13] Galiè N, Humbert M, Vachiery J-L (2016). 2015 ESC/ERS Guidelines for the diagnosis and treatment of pulmonary hypertension: The Joint Task Force for the Diagnosis and Treatment of Pulmonary Hypertension of the European Society of Cardiology (ESC) and the European Respiratory Society (ERS): Endorsed by: Association for European Paediatric and Congenital Cardiology (AEPC), International Society for Heart and Lung Transplantation (ISHLT). Eur Heart J.

[14] Stevens GA, Alkema L, Black RE (2016). Guidelines for Accurate and Transparent Health Estimates Reporting: the GATHER statement. Lancet.

[15] GBD 2021 Demographics Collaborators (2024). Global age-sex-specific mortality, life expectancy, and population estimates in 204 countries and territories and 811 subnational locations, 1950–2021, and the impact of the COVID-19 pandemic: a comprehensive demographic analysis for the Global Burden of Disease Study 2021. Lancet.

[16] GBD 2021 Diseases and Injuries Collaborators (2024). Global incidence, prevalence, years lived with disability (YLDs), disability-adjusted life-years (DALYs), and healthy life expectancy (HALE) for 371 diseases and injuries in 204 countries and territories and 811 subnational locations, 1990–2021: a systematic analysis for the Global Burden of Disease Study 2021. Lancet.

[17] Zheng P, Barber R, Sorensen RJD, Murray CJL, Aravkin AY (2021). Trimmed constrained mixed effects models: formulations and algorithms. J Comp Graph Stat.

[18] Fraisse A, Jais X, Schleich J-M (2010). Characteristics and prospective 2-year follow-up of children with pulmonary arterial hypertension in France. Arch Cardiovasc Dis.

[19] Strange G, Playford D, Stewart S (2012). Pulmonary hypertension: prevalence and mortality in the Armadale echocardiography cohort. Heart.

[20] Kolaitis NA, Zamanian RT, de Jesus Perez VA (2021). Clinical differences and outcomes between methamphetamine-associated and idiopathic pulmonary arterial hypertension in the Pulmonary Hypertension Association Registry. Ann Am Thorac Soc.

[21] Colley DG, Bustinduy AL, Secor WE, King CH (2014). Human schistosomiasis. Lancet.

[22] Jahagirdar D, Walters MK, Novotney A (2021). Global, regional, and national sex-specific burden and control of the HIV epidemic, 1990–2019, for 204 countries and territories: the Global Burden of Diseases Study 2019. Lancet HIV.

[23] GBD 2019 Diseases and Injuries Collaborators (2020). Global burden of 369 diseases and injuries in 204 countries and territories, 1990–2019: a systematic analysis for the Global Burden of Disease Study 2019. Lancet.

[24] Fling C, De Marco T, Kime NA (2023). Regional variation in pulmonary arterial hypertension in the United States: the Pulmonary Hypertension Association Registry. Ann Am Thorac Soc.

[25] Saunders H, Helgeson SA, Abdelrahim A (2022). Comparing diagnosis and treatment of pulmonary hypertension patients at a pulmonary hypertension center versus community centers. Diseases.

[26] Deaño RC, Glassner-Kolmin C, Rubenfire M (2013). Referral of patients with pulmonary hypertension diagnoses to tertiary pulmonary hypertension centers: the multicenter RePHerral study. JAMA Intern Med.

[27] Leary PJ, Lahm T, Melendres-Groves L, Reddy A (2019). Which needle in which haystack? Multisystem care for pulmonary hypertension patients. Ann Am Thorac Soc.

[28] Song Y, Skinner J, Bynum J, Sutherland J, Wennberg JE, Fisher ES (2010). Regional variations in diagnostic practices. N Engl J Med.

[29] D'Agostino A, Guindani P, Scaglione G (2023). Sex- and gender-related aspects in pulmonary hypertension. Heart Fail Clin.

[30] Rich S, Dantzker DR, Ayres SM (1987). Primary pulmonary hypertension. A national prospective study. Ann Intern Med.

[31] Rich S, Chomka E, Hasara L (1989). The prevalence of pulmonary hypertension in the United States. Adult population estimates obtained from measurements of chest roentgenograms from the NHANES II Survey. Chest.

[32] Hoeper MM, Huscher D, Pittrow D (2016). Incidence and prevalence of pulmonary arterial hypertension in Germany. Int J Cardiol.

[33] Hoeper MM, Huscher D, Ghofrani HA (2013). Elderly patients diagnosed with idiopathic pulmonary arterial hypertension: results from the COMPERA registry. Int J Cardiol.

[34] Frost AE, Badesch DB, Barst RJ (2011). The changing picture of patients with pulmonary arterial hypertension in the United States: how REVEAL differs from historic and non-US contemporary registries. Chest.

[35] Jing Z-C, Xu X-Q, Han Z-Y (2007). Registry and survival study in Chinese patients with idiopathic and familial pulmonary arterial hypertension. Chest.

[36] Abenhaim L, Moride Y, Brenot F (1996). Appetite-suppressant drugs and the risk of primary pulmonary hypertension. N Engl J Med.

[37] de Belen E, McConnell JW, Elwing JM (2023). Gaps in the care of pulmonary hypertension: a cross-sectional patient simulation study among practicing cardiologists and pulmonologists. J Am Heart Assoc.

