# Supplementary material for: Global, regional, and national burden of pulmonary arterial hypertension, 1990–2021: a systematic analysis for the Global Burden of Disease Study 2021
Source: Lancet Respir Med. 2025 Jan;13(1):69–79. doi: 10.1016/S2213-2600(24)00295-9 (PMC11698691; doi:10.1016/S2213-2600(24)00295-9)
Supplement: Supplementary appendix 1 [file mmc1.pdf]

THE LANCET
Respiratory Medicine

Supplementary appendix 1

This appendix formed part of the original submission and has been peer reviewed. We post it as supplied by the authors.

Supplement to: GBD 2021 Pulmonary Arterial Hypertension Collaborators. Global, regional, and national burden of pulmonary arterial hypertension, 1990–2021: a systematic analysis for the Global Burden of Disease Study 2021. *Lancet Respir Med* 2024; published online Oct 18. https://doi.org/10.1016/S2213-2600(24)00295-9.

Appendix to “Global, Regional and National Burden of Pulmonary Arterial Hypertension, 1990-2021: a Systematic Analysis for the Global Burden of Disease Study 2021”

This appendix provides additional results and further methodological detail for “Global, Regional and National Burden of Pulmonary Arterial Hypertension, 1990-2021: a Systematic Analysis for the Global Burden of Disease Study 2021”

Portions of this appendix have been reproduced or adapted from:
Naghavi et al. Global burden of 288 causes of death and life expectancy decomposition in 204 countries and territories and 811 subnational locations, 1990-2021: a systematic analysis for the Global Burden of Disease Study 2021.[1]

Ferrari et al. Global incidence, prevalence, years of life lived with disability (YLDs), disability-adjusted life-years (DALYs), and healthy life expectancy (HALE) for 371 diseases and injuries in 204 countries and territories and 811 subnational locations, 1990-2021: a systematic analysis for the Global Burden of Disease Study 2021[2]

Table of Contents

Gather Checklist 3

Appendix Table E1 4

Appendix Figure E1 5

Cause of Death Ensemble Modeling 6

Spatiotemporal Gaussian Process Regression 9

DisMod-MR 2.1 Estimation 13

Data processing 17

Cause of Death Estimation for Pulmonary Arterial Hypertension 18

Nonfatal Burden Estimation for Pulmonary Arterial Hypertension 20

Checklist of information that should be included in new reports of global health estimates

Item #	Checklist item	Reported on page #
**Objectives and funding**		
1	Define the indicator(s), populations (including age, sex, and geographic entities), and time period(s) for which estimates were made.	5
2	List the funding sources for the work.	1
**Data Inputs**		
For all data inputs from multiple sources that are synthesized as part of the study:		
3	Describe how the data were identified and how the data were accessed.	6-7
4	Specify the inclusion and exclusion criteria. Identify all ad-hoc exclusions.	6-7
5	Provide information on all included data sources and their main characteristics. For each data source used, report reference information or contact name/institution, population represented, data collection method, year(s) of data collection, sex and age range, diagnostic criteria or measurement method, and sample size, as relevant.	6-7, appendix
6	Identify and describe any categories of input data that have potentially important biases (e.g., based on characteristics listed in item 5).	6-7
For data inputs that contribute to the analysis but were not synthesized as part of the study:		
7	Describe and give sources for any other data inputs.	N/A
For all data inputs:		
8	Provide all data inputs in a file format from which data can be efficiently extracted (e.g., a spreadsheet rather than a PDF), including all relevant meta-data listed in item 5. For any data inputs that cannot be shared because of ethical or legal reasons, such as third-party ownership, provide a contact name or the name of the institution that retains the right to the data.	7
**Data analysis**		
9	Provide a conceptual overview of the data analysis method. A diagram may be helpful.	6-7, appendix
10	Provide a detailed description of all steps of the analysis, including mathematical formulae. This description should cover, as relevant, data cleaning, data pre-processing, data adjustments and weighting of data sources, and mathematical or statistical model(s).	6-7, appendix
11	Describe how candidate models were evaluated and how the final model(s) were selected.	6-7, appendix
12	Provide the results of an evaluation of model performance, if done, as well as the results of any relevant sensitivity analysis.	6-7, appendix
13	Describe methods for calculating uncertainty of the estimates. State which sources of uncertainty were, and were not, accounted for in the uncertainty analysis.	6-7, appendix
14	State how analytic or statistical source code used to generate estimates can be accessed.	8
**Results and Discussion**		
15	Provide published estimates in a file format from which data can be efficiently extracted.	10
16	Report a quantitative measure of the uncertainty of the estimates (e.g. uncertainty intervals).	8-10
17	Interpret results in light of existing evidence. If updating a previous set of estimates, describe the reasons for changes in estimates.	10-14
18	Discuss limitations of the estimates. Include a discussion of any modelling assumptions or data limitations that affect interpretation of the estimates.	10-14

This checklist should be used in conjunction with the GATHER statement and Explanation and Elaboration document, found on gather-statement.org

Appendix Table E1. Absolute and relative global burden of pulmonary arterial hypertension from1990-2021, including years of life lost (YLLs) and disability-adjusted life years (DALYs)

Total burden across all ages with 95% UI

	YLL			DALY		
Year	Male	Female	Total	Male	Female	Total
1990	366,000 (312,000-421,000)	312,000 (188,000-438,000)	678,000 (525,000-804,000)	370,000 (316,000-425,000)	318,000 (195,000-444,000)	687,000 (535,000-813,000)
1995	351,000 (304,000-402,000)	314,000 (208,000-424,000)	665,000 (552,000-781,000)	355,000 (309,000-407,000)	321,000 (215,000-431,000)	676,000 (562,000-792,000)
2000	331,000 (290,000-385,000)	315,000 (224,000-422,000)	646,000 (550,000-754,000)	336,000 (294,000-389,000)	323,000 (232,000-429,000)	658,000 (563,000-765,000)
2005	326,000 (286,000-371,000)	322,000 (238,000-419,000)	647,000 (557,000-742,000)	331,000 (291,000-376,000)	330,000 (248,000-428,000)	661,000 (572,000-755,000)
2010	351,000 (288,000-397,000)	353,000 (262,000-425,000)	704,000 (596,000-796,000)	356,000 (295,000-402,000)	362,000 (273,000-435,000)	719,000 (612,000-813,000)
2015	336,000 (280,000-381,000)	351,000 (273,000-435,000)	687,000 (588,000-781,000)	342,000 (286,000-388,000)	362,000 (285,000-443,000)	704,000 (606,000-799,000)
2020	298,000 (248,000-346,000)	332,000 (273,000-417,000)	630,000 (452,000-717,000)	305,000 (256,000-352,000)	343,000 (283,000-426,000)	648,000 (562,000-742,000)
2021	293,000 (240,000-343,000)	331,000 (273,000-420,000)	624,000 (536,000-715,000)	300,000 (247,000-348,000)	342,000 (283,000-431,000)	642,000 (552,000-729,000)

Age-standardized rate per 100,000 persons with 95% UI

	YLL			DALY		
Year	Male	Female	Total	Male	Female	Total
1990	13.9 (11.8-16.2)	12.1 (7.49-16.5)	13 (10.6-15.2)	14.1 (11.9-16.4)	12.3 (7.78-16.8)	13.2 (10.8-15.4)
1995	13 (11.2-14.8)	11.6 (7.78-15.5)	12.3 (10.3-14.2)	13.1 (11.3-15)	11.9 (8.05-15.8)	12.5 (10.5-14.4)
2000	11.8 (10.2-13.6)	11 (7.88-14.7)	11.4 (9.74-13.2)	11.9 (10.4-13.8)	11.2 (8.13-15)	11.6 (10-13.4)
2005	10.9 (9.49-12.4)	10.3 (7.67-13.5)	10.6 (9.17-12.1)	11 (9.65-12.5)	10.6 (7.95-13.8)	10.8 (9.42-12.3)
2010	10.9 (8.91-12.3)	10.4 (7.66-12.5)	10.7 (9.05-12.1)	11.1 (9.09-12.6)	10.6 (7.98-12.8)	10.9 (9.31-12.3)
2015	9.7 (8.04-11)	9.47 (7.31-11.7)	9.59 (8.19-10.9)	9.87 (8.22-11.2)	9.73 (7.63-11.9)	9.81 (8.43-11.1)
2020	8.11 (6.73-9.39)	8.28 (6.73-10.4)	8.2 (7.09-9.36)	8.28 (6.94-9.55)	8.53 (7-10.6)	8.42 (7.3-9.63)
2021	7.9 (6.55-9.23)	8.14 (6.66-10.3)	8.03 (6.95-9.21)	8.06 (6.72-9.36)	8.39 (6.92-10.5)	8.24 (7.14-9.39)

Appendix Figure E1. All-ages prevalence and cause-specific mortality for pulmonary arterial hypertension by sex and GBD region in 2021

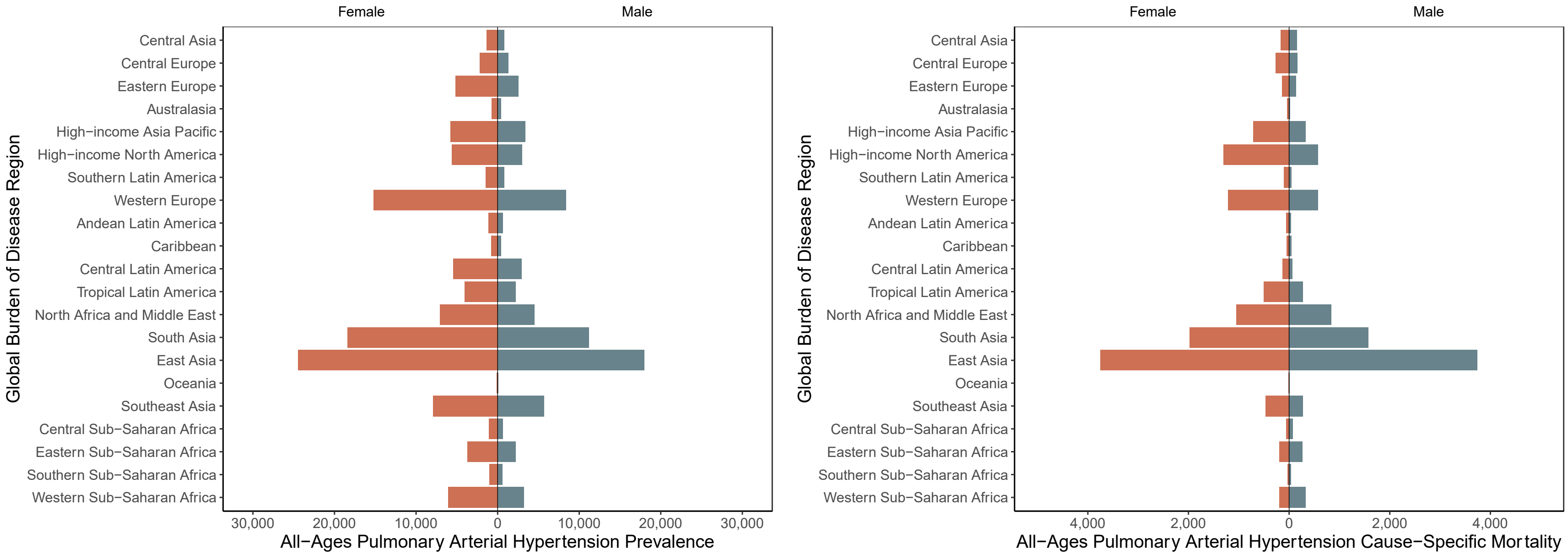

Causes of death ensemble modelling[1]

Overview of methods

Cause of death ensemble modelling (CODEm) is the framework used to model most cause-specific death rates in the GBD.[3] It relies on four key components:

First, all available data are identified and gathered to be used in the modelling process. Although the data may vary in quality, they all contain some signal of the true epidemiological process.

Second, a diverse set of plausible models are developed to capture well-documented associations in the estimates. Using a wide variety of individual models to create an ensemble predictive model has been shown to outperform techniques using only a single model both in CoD estimation and in more general prediction applications.[3–5]

Third, the out-of-sample predictive validity is assessed for all individual models, which are then ranked for use in the ensemble modelling stage.

Finally, differently weighted combinations of individual models are evaluated to select the ensemble model with the highest out-of-sample predictive validity.

For some causes (eg, lower respiratory infections), evidence exists that the relationship between covariates and death rates might differ between children and adults. Separate models are therefore run for different age ranges, when applicable. Additionally, separate models are developed for countries with extensive, complete, and representative VR for every cause to ensure that uncertainty can better reflect the more complete data in these locations.

In order to ensure the addition of subnational locations is not driving changes in estimates, in GBD 2021, we run a global model that excludes data from non-standard locations; the resulting covariate betas are then used as priors for the true global model.

In addition to CoD modelling, we also estimate fatal discontinuities. Fatal discontinuities are events that are stochastic in nature, that cannot be modelled because they do not have a predictable time trend. The fatal discontinuities by cause are aggregated by age and sex and added to the estimated number of deaths in CoD modelling for those causes during CoDCorrect.

Model pool development

Because many factors may co-vary with any given CoD, a range of plausible statistical models are developed for each cause. In the CODEm framework, four families of statistical models are used: linear mixed effects regression (LMER) models of the natural log of the cause-specific death rate, LMER models of the logit of the cause fraction, spatiotemporal Gaussian process regression (ST-GPR) models of the natural logarithm of the cause-specific death rate, and ST-GPR models of the logit of the cause fraction (see the 2x2 table in Foreman et al).[3] For more on ST-GPR, please see the following section. For each family of models, all plausible relationships between covariates and the response variable are identified. Because all possible combinations of selected covariates are considered for each family of models, multi-collinearity between covariates may produce implausible signs on coefficients or unstable coefficients. Each combination is therefore tested for statistical significance (covariate coefficients must have a coefficient with p-value <0·05) and plausibility (the coefficients must have the directions

expected on the basis of the literature). Only covariate combinations meeting these criteria are retained. This selection process is run for both cause fractions and death rates, then ST-GPR and LMER-only models are created for each set of covariates. For a detailed explanation of the covariate selection algorithm, see Foreman et al.[3]

Data variance estimation

The families of models that go through ST-GPR described in Section 4.1.2 incorporate information about data variance. The main inputs for a Gaussian process regression (GPR) are a mean function, a covariance function, and data variance for each datapoint. These inputs are described in detail in Foreman et al.[3] For GBD 2019, we updated this calculation to incorporate garbage code redistribution uncertainty.

Three components of data variance are now used in CODEm: sampling variance, non-sampling variance, and garbage code redistribution variance. The computation of sampling variance and non-sampling variance has not changed since previous iterations of the GBD and is also described in Foreman et al.[3] Garbage code redistribution variance is computed in the CoD database process described elsewhere. Since variance is additive, we calculate total data variance as the sum of sampling variance, non-sampling variance, and redistribution variance. Increased data variance in GPR results in the GPR draws not following the datapoint as closely.

Testing model pool on 15% sample

The performance of all models (individual and ensemble) is evaluated by means of out-of-sample predictive validity tests. 30% of the data are randomly excluded from the initial model fits. These individual model fits are evaluated and ranked by using half of the excluded data (15% of the total), then used to construct the ensembles on the basis of their performance. Data are held out from the analysis on the basis of the cause-specific missingness patterns for ages and years across locations. Out-of-sample predictive validity testing is repeated 20 times for each model, which has been shown to produce stable results.[3] These performance tests include the root mean square error (RMSE) for the log of the cause-specific death rate, the direction of the predicted versus actual trend in the data, and the coverage of the predicted 95% UI.

Ensemble development and testing

The component models are weighted on the basis of their predictive validity rank to determine their contribution to the ensemble estimate. The relative weights are determined both by the model ranks and by a parameter ψ, whose value determines how quickly the weights taper off as rank decreases. The distribution of ψ is described in more detail in Foreman et al.[3] A set of ensemble models is then created by using the weights constructed from the combinations of ranks and ψ values. These ensembles are tested by using the predictive validity metrics described in Section 4.1.4 on the remaining 15% of the data, and the ensemble with the best performance in out-of-sample trend and RMSE is chosen as the final model.

Final estimation

Once a weighting scheme has been chosen, 1000 draws are created for the final ensemble, and the number of draws contributed by each model is proportional to its weight. The mean of the draws is used as the final estimate for the CODEm process, and a 95% UI is created from the 0·025 and 0·975 quantiles of the draws. The validity of the UI can be checked via its coverage of the out-of-sample data; ideally,

the 95% UI would capture 95% of these data. Higher coverage suggests that the UIs are too large, and lower coverage suggests overfitting.

Model-specific covariates

Modellers select covariates to be used in CODEm, but those covariates may not be significant or in the direction specified during the covariate selection step of CODEm and will therefore not be used in the model. These covariates are listed with a '—' for number of draws. Additionally, covariates may be selected by CODEm but only exist in submodels that perform poorly and may end up with zero draws included in the final ensemble. Finally, all other covariates are listed with the number of draws in the final ensemble from submodels that had the covariate.

Spatiotemporal Gaussian process regression (ST-GPR) modelling[2]

Overview

The approach is a stochastic modelling technique that is designed to detect signals amidst noisy data. It also serves as a powerful tool for interpolating non-linear trends.[6,7] Unlike classical linear models that assume that the trend underlying data follows a definitive functional form, GPR assumes that the specific trend of interest follows a Gaussian process, which is defined by a mean function $m(\cdot)$ and a covariance function $Cov(\cdot)$. For example, let $p_{c,a,s,t}$ be the prevalence, in normal, log, or logit space, observed in country *c*, for age group *a*, and sex *s* at time t:

$$\left(p_{c,a,s,t}\right) = g_{c,a,s}(t) + \epsilon_{c,a,s,t}$$

where

$$\epsilon_{c,a,s,t} \sim Normal\left(0, \sigma_p^2\ \right),$$
$$g_{c,a,s}(t) \sim GP\left(m_{c,a,s}(t), Cov\left(g_{c,a,s}(t)\right)\right).$$

The derivation of the mean and covariance functions, $m_{c,a,s}(t)$ and $Cov\left(g_{c,a,s}(t)\right)$, along with a more detailed description of the error variance (σ_p^2), is described below.

Estimating mean functions

We estimated mean functions by using a two-step approach. To be more specific, $m_{c,a,s}(t)$ can be expressed, depending on the prevalence transformation, as:

$$log\left(p_{c,a,s}(t)\right) = X_{c,a,s}\beta + h(r_{c,a,s,t})$$
$$logit\left(p_{c,a,s}(t)\right) = X_{c,a,s}\beta + h(r_{c,a,s,t})$$
$$p_{c,a,s}(t) = X_{c,a,s}\beta + h(r_{c,a,s,t})$$

where $X\beta$ is the summation of the components of a hierarchical mixed-effects linear regression, including the intercept and the product of covariates with their corresponding fixed-effect coefficients. Some models were run as hierarchical mixed-effects linear regressions with random effects on the levels of the location hierarchy. For most mixed-effects models, random effects were only used in the fit, not in the prediction. The second part of the equation, $h(r_{c,a,s,t})$, is a smoothing function for the residuals, $r_{c,a,s,t}$, derived from the linear model.[8]

Although the linear component captures general trends over time, much of the data variability may still not be adequately accounted for. To address this, we fit a locally weighted polynomial regression (locally estimated scatterplot smoothing, or LOESS) function $h(r_{c,a,s,t})$ to systematically estimate this residual variability by borrowing strength across time, age, and space patterns (the spatiotemporal component of ST-GPR).[9,10] The time adjustment parameter, defined by λ , aims to borrow strength from neighboring time points (ie, the prevalence in this year is highly correlated with prevalence in the previous year but less so further back in time). The age-adjustment parameter, defined by ω, borrows strength from data in neighboring age groups. The space-adjustment parameter, defined by ξ, aims to borrow strength across the hierarchy of geographical locations. The spatial and temporal weights are combined into a single space-time weight to allow the amount of spatial weight given to a particular point $r_{c,a,s,t}$ to fluctuate given the data availability at each time *t* and location-level *l* in the location hierarchy.

Let $w_{c,a,s,t}$ be the final weight assigned to observation $r_{c,a,s,t}$ with reference to a focal observation r_{c_0,a_0,s_0,t_0}. We first generated a temporal weight $t.w_{c,a,s,t}$ for smoothing over time, which was based on the scaled distance along the time dimension of the two observation:[9]

$$t.w_{c,a,s,t} = \frac{1}{e^{\lambda|t-t_0|}}$$

Next, we generated a spatial weight to smooth over geography. Specifically, we defined a geospatial relationship by categorizing data based on the GBD location hierarchy (table 1). *zeta* acts as a scalar on a given datapoint given its proximity to the target location:

$$t.w_{c,a,s,t} = \zeta^{|c-c_0|}$$

For example, estimating a country, would use the following weighting scheme:

- Country data: ζ^0 = 1
- Regional data not from the country being estimated: ζ^1
- Data from other regions in the same super region: ξ^2
- Global data from other super regions: ζ^3

Under the spatial weighting specification, typical values of ζ range from [0.001, 0.2], where ζ can be interpreted as the amount to downweight regional datapoints compared to country datapoints for a given estimating country. For example, for a given datapoint $r_{c,a,s,t}$ and ζ = 0.01, a datapoint not within country *c* but within the same region *r* as $r_{c,a,s,t}$ would be assigned $\frac{1}{100}$ the weight of a datapoint within the country.

The spatial and temporal weights were then multiplied and summed across each level of the location hierarchy and normalised for each time period *t* . This procedure allowed the space-time weight to implicitly take into account the amount of data available at the country vs. region vs. super-region level and attribute spatial weight accordingly.

Given a normalisation constant,

$$K_i = \sum_{c \epsilon C} s.w_{c,t} * t.w_{c.t} + \sum_{c \epsilon R} s.w_{c,t} * t.w_{c.t} + \sum_{c \epsilon SR} s.w_{c,t} * t.w_{c.t}$$

the final space-time weight would then equal

$$w'_{c,a,s,t} = \frac{s.w_{c,t} * t.w_{c,t}}{K_i}$$

Finally, we calculated the weight $w''_{c,a,s,t}$ to smooth over age, which is based on a distance along the age dimension of two observations. For a point between the age a of the observation $r_{c,a,s,t}$ and a focal observation r_{c_0,a_0,s_0,t_0}, the weight is defined as follows:

$$w''_{c,a,s,t} = \frac{1}{e^{\omega|a-a_0|}}$$

The final weights were then computed by simply multiplying the space-time weights and age weights and normalising so all weights for a given time period *t* sum to 1. A full derivation of weights for each category, assuming the location being estimated was a country, follows:

1) If the observation $r_{c,t}$ belongs to the same country c_0 of the focal observation r_{c_0,t_0}:

$$w_{c,a,s,t} = \frac{(w'_{c,a,s,t} w''_{c,a,s,t})}{\sum_{c=c_0}(w'_{c,a,s,t} w''_{c,a,s,t})} \qquad \forall c = c_0$$

2) If the observation $r_{c,t}$ belongs to a different country than the focal observation r_{c_0,t_0}, but both belong to the same region *R*:

$$w_{c,a,s,t} = \frac{(w'_{c,a,s,t}w''_{c,a,s,t})}{\sum_{c\neq c_0}(w'_{c,a,s,t}w''_{c,a,s,t})} \qquad \forall c \neq c_0 \cap\ R[c] = R[c_0]$$

3) If the observation $r_{c,t}$ belongs to the same super region *SR* but to both a different country c_0 and a different region $R[c_0]$ than the focal observation r_{c_0,t_0}:

$$w_{c,a,s,t} = \frac{(w'_{c,a,s,t}w''_{c,a,s,t})}{\sum_{c\neq c_0}(w'_{c,a,s,t}w''_{c,a,s,t})} \qquad \forall c \neq c_0 \cap\ R[c] \neq R[c_0] \cap\ SR[c] = SR[c_0]$$

4) If the observation $r_{c,t}$ is from a different super region than the focal observation r_{c_0,t_0}(Ie, all other data currently not receiving a weight):

$$w_{c,a,s,t} = \frac{(w'_{c,a,s,t}w''_{c,a,s,t})}{\sum_{c\neq c_0}(w'_{c,a,s,t}w''_{c,a,s,t})} \qquad \forall c \neq c_0 \cap\ R[c] \neq R[c_0] \cap\ SR[c] \neq SR[c_0]$$

Observations could be downweighted by a factor of 0.1, usually because they were not geographically representative at the unit of estimation. Details of reasons for downweighting can be found in cause-specific modeling summaries. The final weights were then normalised such that the sum of weights across age, time, and geographic hierarchy for a reference group was 1.

Estimating error variance

σ_p^2 represents the error variance in normal or transformed space including the sampling variance of the estimates and prediction error from any crosswalks performed.

First, variance was systematically imputed if the data extraction did not include any measure of uncertainty. When some sample sizes for data were available, missing sample sizes were imputed as the 5th percentile of available sample sizes. Missing variances were then calculated as $\sigma_p^2 = \frac{p*(1-p)}{n}$ for proportions or were predicted from the mean by using a regression for continuous values. When sample sizes were entirely missing and could not be imputed, the 95th percentile of available variances at the most granular geographic level (ie, first country, then region, etc.) were used to impute missing variances. For proportions where *p*n* or *(1-p)*n* is <20, variance was replaced by using the Wilson Interval Score method.

Next, if prevalence was modelled as a log transformation, the error variance was transformed into log-space by using the delta method approximation as follows:

$$\sigma_p^2 \cong \frac{\sigma_{p\prime}^2}{p_{c,a,s,t}^2}$$

where $\sigma_{p\prime}^2$, represents the error variance in normal space. If prevalence was modelled as a logit transformation, the error variance was transformed into logit-space by using the delta method approximation as follows:

$$\sigma_p^2 \cong \frac{\sigma_{p\prime}^2}{(p_{c,a,s,t} * (1-p_{c,a,s,t}))^2}$$

Finally, prior to GPR, an approximation of non-sampling variance was added to the error variance. Calculations of non-sampling variance were done on normal-space variances. Non-sampling variance was calculated as the variance of inverse-variance weighted residuals from the space-time estimate at a given location-level hierarchy. If there were <10 data points at a given level of the location hierarchy, the non-sampling variance was replaced with that of the next highest geography level with >10 data points.

Estimating the covariance function

The final input into GPR is the covariance function, which defines the shape and distribution of the trends. Here, we have chosen the Matern-Euclidian covariance function, which offers the flexibility to model a wide spectrum of trends with varying degrees of smoothness. The function is defined as follows:

$$M(t,t') = \sigma^2 \frac{2^{1-v}}{\Gamma(v)} \left(\frac{d(t,t')\sqrt{2v}}{l}\right)^v K_v\left(\frac{d(t,t')\sqrt{2v}}{l}\right)$$

where $d(\cdot)$is a distance function; σ^2, v, l, and K_v are hyperparameters of the covariance function—specifically σ^2 is the marginal variance, v is the smoothness parameter that defines the differentiability of the function, l is the length scale, which roughly defines the distance between which two points become uncorrelated, and K_v is the Bessel function. We approximated σ^2 by taking the normalised median absolute deviation $MADN(r_c')$ of the difference, which is the normalised absolute deviation of the difference of the first-stage linear regression estimate from the second-stage spatiotemporal smoothing step for each country. We then took the mean of these country-level MADN estimates for all countries with 10+ country-years of data to ensure that differences between first- and second-stage estimates had sufficient data to truly convey meaningful information on model uncertainty. We used the parameter specification $v = 2$ for all models. The scale parameter l used for each cause is reported in appendix sections 3.4 and 4.12.

Prediction using GPR

We integrated over $g_{c,t}(t_*)$ to predict a full time series for country c, age a, sex s, and prediction time t_*as follows:

$$p_{c,a,s}(t_*) \sim N\left(m_{c,a,s,t}(t_*), \sigma_p^2 I + Cov\left(g_{c,a,s,t}(t_*)\right)\right)$$

Random draws of 1000 samples were obtained from the distributions above for every country for a given indicator. The final estimated mean for each country was the mean of the draws. In addition, 95% UIs were calculated by taking the 2.5 and 97.5 percentile of the sample distribution. The linear modelling process was implemented by using the lmer4 package in R, and the ST-GPR analysis was implemented through the PyMC2 package in Python.

Subnational scaling and aggregation

To ensure internal consistency of the estimates between countries and their respective subnational locations, national estimates were either created by population-weighted aggregation or subnational estimates were adjusted by population-weighted scaling to the national estimates, depending on the data coverage of a given country compared to that of its subnational locations. For example, if data coverage was better at the national level than at its corresponding subnational locations for a given country and cause across age, sex, and time, estimates were rescaled to be consistent with the national level. Conversely, if data coverage was better at the subnational level, estimates for its parent country were generated through population-weighted aggregation of subnational estimates.

Estimates can also be scaled within logit space. Scaling in logit space ensures that subnational estimates of proportion models do not exceed one after being rescaled to the national estimate.

DisMod-MR 2.1 estimation[2]

Estimation of sequelae and causes

The most extensively used estimation method is the Bayesian meta-regression method DisMod-MR 2.1. For some causes, such as HIV/AIDS or measles, disease-specific natural history models have been used for which the underlying three-state model in DisMod-MR 2.1 (susceptible, cases, dead) is insufficient to capture the complexity of a disease process. For some diseases with a range of sequelae differentiated by severity, such as COPD or diabetes mellitus, DisMod-MR 2.1 was used to meta-analyse the data on overall prevalence with separate DisMod-MR 2.1 models of the proportions of cases with different severity levels or sequelae. Likewise, DisMod-MR 2.1 was used to meta-analyse data on the proportions of liver cancer and cirrhosis due to underlying aetiologies such as hepatitis B, hepatitis C, and alcohol use disorders.

DisMod-MR 2.1 description

Until GBD 2010, non-fatal estimates in burden of disease assessments were based on a single data source on prevalence, incidence, remission, or a mortality risk selected by the researcher as most relevant to a particular location and time. For GBD 2010, we set a more ambitious goal: to evaluate all available information on a disease that passes a minimum quality standard. That required a different analytical tool that would be able to pool disparate information presented for varying age groupings and from data sources by using different case definitions. The DisMod-MR 1.0 tool used in GBD 2010 evaluated and pooled all available data, adjusted data for systematic bias associated with case ascertainment methods that varied from the reference and produced estimates by world regions with UIs by using Bayesian statistical methods.

For GBD 2013, the improved DisMod-MR 2.0 increased computational speed, which allowed computations to be consistent between all disease parameters at the country rather than the region level. The hundred-fold increase in speed of DisMod-MR 2.0 was partly due to a more efficient rewrite of the code in C++, but also due to switching to a model specification of log rates rather than a negative binomial model used in DisMod-MR 1.0. In cross-validation tests, the log rates specification worked as well as or better than the negative binomial specification.[11] The sequence of estimation occurs at five levels: global, super-region, region, country and, where applicable, subnational location. The super-region priors are generated at the global level with mixed-effects, non-linear regression by using all available data; the super-region fit, in turn, informs the region fit, and so on down the cascade. Analysts can choose to branch the cascade in terms of time and sex at different levels depending on data density. The default used in most models is to branch by sex after the global fit but to retain all years of data until the lowest level in the cascade is reached.

The computational engine is limited to three levels of random effects; we differentiate estimates at the super-region, region, and country level. In GBD 2013, the subnational units of China, the United Kingdom and Mexico were treated as “countries” to enable a random effect to be estimated for every location with contributing data. However, the lack of a hierarchy between country and subnational units meant that the fit to country data contributed as much to the estimation of a subnational unit as the fits for all other countries in the region. We found inconsistency between the country fit and the aggregation of subnational estimates when the country’s epidemiology varied from the average of the region. Adding an additional level of random effects required a prohibitively comprehensive rewrite of the underlying DisMod-MR engine. Instead, we added a fifth layer to the cascade, with subnational estimation informed by the country fit and country covariates, plus an adjustment based on the average

of the residuals between the subnational location's available data and its prior. This technique mimicked the impact of a random effect on estimates among subnationals.

In GBD 2015, we also improved how country covariates differentiate non-fatal estimates for diseases with sparse data. The coefficients for country covariates are re-estimated at each level of the cascade. For a given location, country coefficients are calculated by using both data and prior information available for that location. In the absence of data, the coefficient of its parent location is used to utilise the predictive power of our covariates in data-sparse situations.

For GBD 2016, the computational engine (DisMod-MR 2.1) remained substantively unchanged from GBD 2015. We updated the age prediction sets to include age groups 80–84 years, 85–89 years, 90–94 years, and 95 years and older to comply with changes across all functional areas of the GBD.

In GBD 2017, we continued to use DisMod-MR 2.1 because no substantial changes were made. Updates to computation include extending the terminal prediction year to 2017 and additional subnational units in Ethiopia, Iran, New Zealand, Norway, and the Russian Federation.
In GBD 2019 and 2021, no substantial changes were made to DisMod-MR 2.1, but we made more substantial changes to how we use the tool. First, we added the years 2019, 2020, and 2021 as additional years of estimation. Second, we also included the option again to have random effects on cause-specific mortality rates (CSMR) and EMR. This functionality had been dropped a couple of GBD rounds earlier. Third, as we did all our adjustments for alternative case definitions and study methods as well as adjustments to combined-sex data points prior to entering data into DisMod-MR 2.1, we no longer used the functionality in DisMod-MR 2.1 to estimate coefficients for study and sex covariates. Fourth, based on simulation testing conducted in GBD 2019 we found that coverage improved, and errors reduced when passing down priors with a wider setting of minimum coefficient of variation (which determines the uncertainty around priors and hence how 'informative' the priors are) than had generally been used in past GBD iterations. We settled on a default value of 0.8 where in the past values of 0.4 or less had been more commonly used. We made some exceptions for highly prevalent conditions where a lower minimum coefficient of variation (CV) setting achieved the task of making priors less informative, but not completely uninformative.

In GBD 2017 and 2019 GBD rounds we calculated priors on excess mortality and entered these as data points by matching sex-specific prevalence data with an age width of 20 or less with the corresponding CSMR for the same location and year. Forstability, we excluded calculation of EMR for prevalence data points of less than 1 in a million. EMR is simply calculated as CSMR divided by prevalence. As with previous GBD years, for diseases with an average duration of less than a year (as indicated by a setting of remission greater than one), we ran an initial global model to get an equivalent prevalence and used the following formula to calculate EMR:

$$EMR = {CSMR * (remission + (ACMR - CSMR) + EMR\_pred)}/{incidence}$$

where,

ACMR is the all-cause mortality rate
EMR_pred is the EMR fit from an initial global DisMod model

Despite using the log of LDI or the HAQ Index as a covariate with a prior that the coefficient had to be negative, we found many disease models with an implausible distribution of mortality to prevalence (or incidence) ratios implying lower case fatality in locations with lower HAQ Index than in countries with higher HAQ Index. This likely signals an inconsistency between fatal and non-fatal data inputs. For GBD 2019, we decided to run regressions on EMR data (calculated as described above) first using MR-BRT with HAQ Index as a predictor. In general, we tend to think that CSMR estimates are more robust than non-fatal data because of much greater data availability and a lesser task in adjusting cause death data for garbage coding than the complex task of adjusting non-fatal data sources for alternative case definitions and study methods. To indicate that we would reduce the random effects on EMR and the minimum coefficient of variation for priors on EMR being created at each next level down the cascade. However, there were exceptions. For drug use disorders, the risk of overdose deaths is less a function of a country's quality of health services but driven more by the availability of harm reduction strategies, such as opioid substitution therapy, and the availability of highly potent opioids such as fentanyl, which have been an important contributor to the large increase in overdose deaths in the USA in the last decade. We settled on a model for opioid use disorder with wider random effects and higher minimum coefficient of variation to give less emphasis on CSMR when enforcing consistency with prevalence data. In a next round, we will work to find covariates that are more relevant to drug overdose deaths such as a grading of harm reduction strategies by country and over time. In the case of COPD, we noted that following the data on CSMR and EMR led to large increases in prevalence estimates in east Asia, Oceania and, to a lesser extent, south Asia. In the oldest age groups, prevalence estimates would be higher than the prevalence data for these locations and reach a level of close to 80% in the oldest age groups. In these locations, we will pay attention to how garbage codes are being redistributed onto COPD in the next round of GBD.

DisMod-MR 2.1 likelihood estimation

Analysts have the choice of using a Gaussian, log-Gaussian, Laplace, or Log-Laplace likelihood function in DisMod-MR 2.1. The default log-Gaussian equation for the data likelihood is

$$-log\left[p\left(y_j|\Phi\right)\right] = \log\left(\sqrt{2\pi}\right) + \log\left(\delta_j + s_j\right) + \frac{1}{2}\left(\frac{\log\left(a_j + \eta_j\right) - \log\left(m_j + \eta_j\right)}{\delta_j + s_j}\right)^2$$

Where,

y_j is a "measurement value" (ie, data point)

Φ denotes all model random variables

η_j is the offset value, *eta*, for a particular "integrand" (prevalence, incidence, remission, excess mortality rate, with-condition mortality rate, cause-specific mortality rate, relative risk, or standardised mortality ratio)

a_j is the adjusted measurement for data point j, defined by

$$a_j = e^{(-u_j - c_j)} y_j$$

Where:

u_j is the total "area effect" (ie, the sum of the random effects at three levels of the cascade: super-region, region and country) and

c_j is the total covariate effect (ie, the mean combined fixed effects for sex, study level, and country level covariates), defined by

$$c_j = \sum_{k=0}^{K[I(j)]-1} \beta_{I(j),k} \hat{X}_{k,j}$$

with SD

$$s_j = \sum_{l=0}^{L[I(j)]-1} \zeta_{I(j),l} \hat{Z}_{k,j}$$

Where:

k denotes the mean value of each data point in relation to a covariate (also called x-covariate)

$I(j)$ denotes a data point for a particular integrand, j

$\beta_{I(j),k}$ is the multiplier of the kth x-covariate for the ith integrand

$\hat{X}_{k,j}$ is the covariate value corresponding to the data point j for covariate k;

l denotes the SD of each data point in relation to a covariate (also called z-covariate)

$\zeta_{I(j),k}$ is the multiplier of the lth z-covariate for the ith integrand

δ_j is the SD for adjusted measurement j, defined by:

$$\delta_j = log\left[y_j + e^{(-u_j - c_j)}\eta_j + c_j\right] - log\left[y_j + e^{(-u_j - c_j)}\eta_j\right]$$

Where:

m_j denotes the model for the jth measurement, not counting effects or measurement noise, and defined by:

$$m_j = \frac{1}{B(j) - A(j)} \int_{A(j)}^{B(j)} I_j(\text{a})\ \text{da}$$

Where:

$A(j)$ is the lower bound of the age range for a data point

$B(j)$ is the upper bound of the age range for a data point

I_j denotes the function of age corresponding to the integrand for data point j

Data Adjustments[2]

Age sex splitting

Before running a full compartmental model to generate estimates for incidence and prevalence, we ran a Dismod-MR 2.1 model with data disaggregated by age to estimate countries' age-pattern and then applied the estimated age-pattern to split aggregated all-age data into 10-year age groups. This procedure was done by calculating a constant, k, which was the ratio of the aggregated all-age data point, $\mu_{all\,age}$, to the all-age estimated utilisation rate from the DisMod-MR 2.1 model, $\widehat{\mu_d}$

$$k = \frac{\mu_{all\,age}}{\widehat{\mu_d}}$$

The constant, k, was then multiplied by age-specific utilisation rates from the DisMod-MR 2.1 model. Observation-specific uncertainty and uncertainty from the estimated age-pattern were both propagated into the uncertainty for a given post-splitting data point. The split data were then incorporated into the final DisMod-MR 2.1 model.

Cause of death estimation for pulmonary arterial hypertension

Flowchart

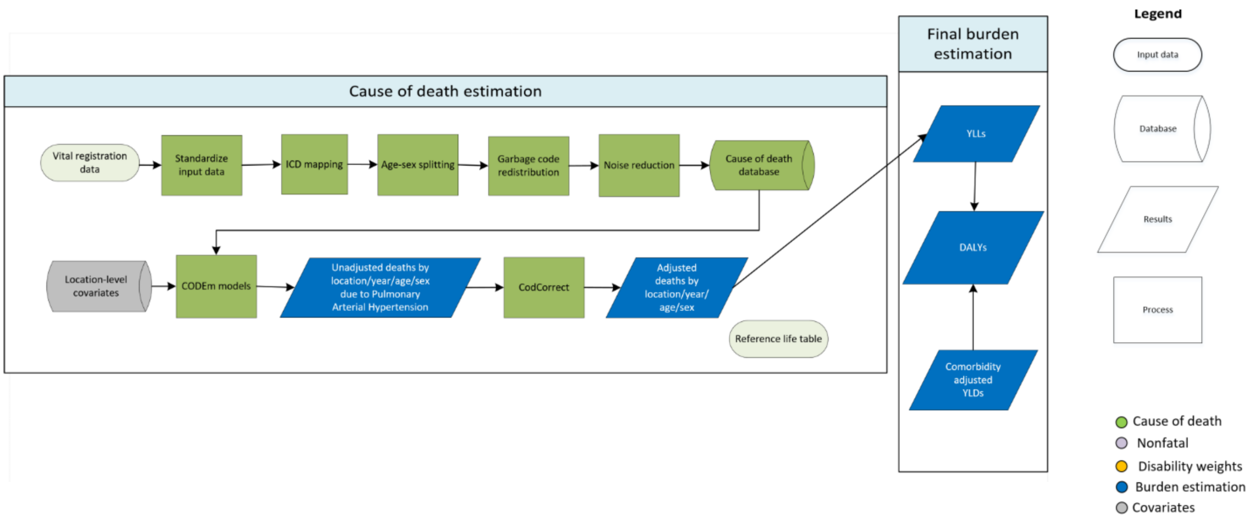

Input Data and Methodological Summary for Pulmonary Arterial Hypertension

Input data

Vital registration data were used to model pulmonary arterial hypertension. ICD codes 416.0 (ICD-9) and I27.0 (ICD-10) were used. We did not include deaths recorded under the ICD-8 coding system, or tabulated data where causes are reported in aggregate, which do not have sufficient detail to distinguish PAH from other pulmonary diseases.

These codes were selected after reviewing clinical guidelines, available ICD codes, and analyses of ICD-coded data reported in the literature. These analyses have found large changes that correspond to the shift from ICD9 to ICD10 and a subsequent update to the ICD10 system.[12,13] It is more plausible that these differences are based on changes in coding systems and coding practices rather than changes in the burden of disease itself.

The ICD10 system included more specific definitions that were intended to, among other things, improve the ability of the coding system to accurately identify PAH as a distinct entity separate from other types of pulmonary hypertension. In 2003, the ICD10 system was further updated in an effort to improve disease classification for pulmonary hypertension. In the ICD10 system, PAH maps to the code I27.0 (Primary pulmonary hypertension), while the other pulmonary hypertension groups map to I27.2 (Other secondary pulmonary hypertension). However, I27.2 was not used prior to 2003 in any location; the first year of use varied by country. When evaluating patterns of mortality, we observed an abrupt shift for many locations which corresponds to the start of deaths being coded to I27.2 in that location.

A highly likely explanation was that before the regular use of I27.2, non-PAH types of pulmonary hypertension were often erroneously coded to I27.0. We thus concluded that the most accurate estimates of PAH burden should be based on deaths coded to I27.0 after the introduction of I27.2.
As a result of this analysis, we excluded all data prior the first use of I27.2 for locations with large changes. For locations where the introduction of I27.2 had no noticeable impact, we included all ICD10-

coded data. Where there were differences between ICD9- and ICD10-coded deaths, we excluded the years where the ICD9 system was used.

Examples of the different patterns are:

1) In Australia the introduction of I27.2 resulted in a large change in the mortality beginning in 2005 and the differences between the ICD9 coded deaths and the I27.0 deaths from 2006 on were likely due to differences between coding systems rather than reflective of true differences in mortality.
2) In Germany the introduction of I27.2 had no impact on the pattern of deaths coded to I27.0 and the deaths coded in the years when the ICD9 system was in use were reasonably consistent with the I27.0 coded deaths.

Modelling strategy

We used a standard CODEm approach to model deaths from pulmonary arterial hypertension. The covariates used along with their transformations, importance levels, and imposed directions are reported by cause in the tables below. Schistosomiasis and HIV were chosen as covariates because these diseases are underlying causes of PAH, and they are major drivers of PAH mortality in some locations. SDI and HAQi were assigned a negative direction to reflect how treatment, screening, and medication can lower mortality of PAH in locations with high SDI or HAQi values. For GBD 2020, we switched from an ensemble of exponential counts models to an ensemble of spacetime models that use exponential smoothing. This improved model fit and reduced uncertainty of estimates for PAH. In addition, we updated our approach to noise reduction of the cause of death data so that stochastic time series of data would resemble patterns seen at regional levels more closely. Additional details on the updates to noise reduction can be found in the cause of death methods section of the appendix.

Table 1. Covariates used in Pulmonary Arterial Hypertension mortality modelling

Level	Covariate	Direction
1	Prevalence of Schistosomiasis	1
	Summary Exposure Value (SEV), HIV	1
	Socio-demographic Index	-1
	Healthcare access and quality index	-1

Nonfatal health outcome burden estimation for pulmonary arterial hypertension

Flowchart

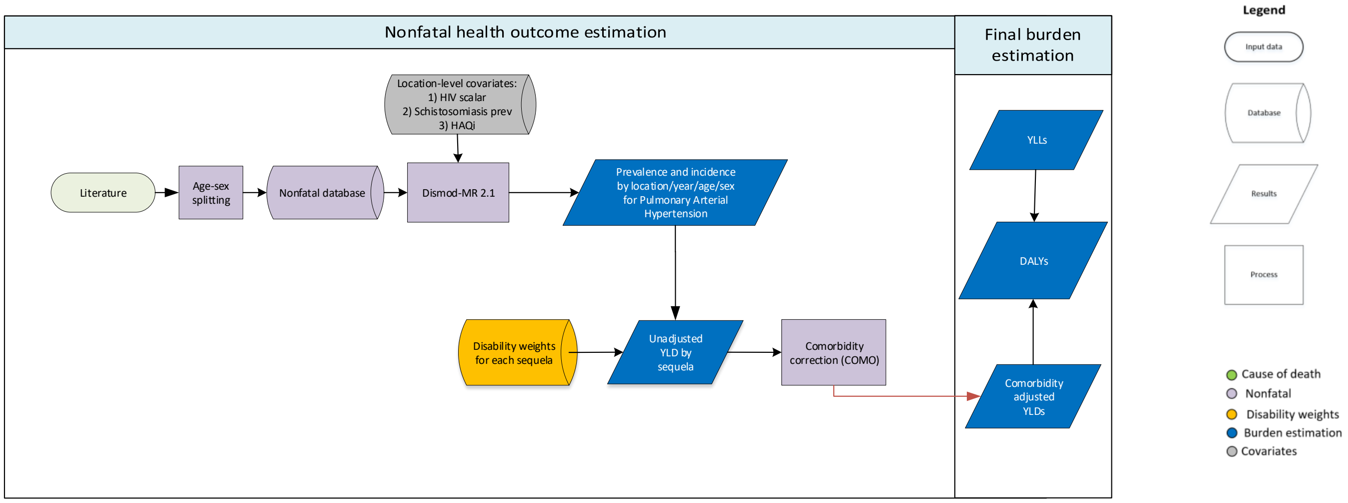

Input data and methodological summary for pulmonary arterial hypertension

Case definition

Pulmonary arterial hypertension (PAH) is a vascular disease in which remodelling of the pulmonary arteries leads to high pulmonary pressures, increased vascular resistance, and eventual right heart dysfunction. It is a form of pulmonary hypertension (PH), characterised by high pressures in the pulmonary system; PAH is consistent with WHO Group 1 pulmonary hypertension (Figure 1)[14]. We restrict our case definition to PAH or Group 1 PH, as other forms of PH are captured in other GBD causes.

The GBD case definition of PAH is clinically diagnosed pulmonary arterial hypertension, with supporting diagnostic evidence either via right heart catheterisation or echocardiogram. We include PAH identified through ICD codes if the study authors have confirmed the diagnosis by reviewing medical records for results from catheterisation or echocardiography. All other forms of pulmonary hypertension are excluded from this cause.

Figure 1: WHO classification of pulmonary hypertension groups 1-5

WHO Classification	Description
Group 1	Pulmonary Arterial Hypertension (PAH)
Group 2	Pulmonary hypertension due to left heart disease
Group 3	Pulmonary hypertension due to lung disease
Group 4	Pulmonary hypertension due to thromboembolic disease
Group 5	Pulmonary hypertension with unclear mechanism

Input data

A systematic review for incidence, prevalence, mortality, and aetiological breakdown for pulmonary arterial hypertension was conducted for GBD 2019 and updated for GBD 2021.

We searched the Global Index Medicus, which indexes PubMed as well as several international journals, on 11/13/2018 with the following string: tw:("pulmonary arterial hypertension") OR tw:("pulmonary artery hypertension") OR tw:("primary pulmonary hypertension") OR tw:("group 1 pulmonary hypertension") OR tw:("group one pulmonary hypertension")) AND (tw:(epidemiology) OR tw:("prevalent cases") OR tw:(prevalence) OR tw:("incident cases") OR tw:(incidence) OR tw:("standardized mortality ratio") OR tw:("case fatality") OR tw:("relative risk of death") OR tw:("excess mortality") OR tw:(survival)) AND NOT (tw:(rats) OR tw:(mice) OR tw:(dogs) OR tw:(apes) OR tw:(monkeys) OR tw:(chickens) OR tw:(pigs) OR tw:(sheep)).

Since the original search, GIM has removed PubMed from its indexing; to account for this we searched PubMed independently for results from 2018–2020 and de-duplicated the results in the final count. We searched PubMed with the following string: ("pulmonary arterial hypertension"[Title] OR "pulmonary arterial hypertension"[Abstract] OR "pulmonary artery hypertension"[Title] OR "pulmonary artery hypertension"[Abstract] OR "primary pulmonary hypertension"[Title] OR "primary pulmonary hypertension"[Abstract] OR "group 1 pulmonary hypertension"[Title] OR "group 1 pulmonary hypertension"[Abstract] OR "group one pulmonary hypertension"[Title] OR "group one pulmonary hypertension"[Abstract]) AND ("epidemiology"[Abstract] OR "prevalent cases"[Abstract] OR "prevalence"[Abstract] OR "incident cases"[Abstract] OR "incidence"[Abstract] OR "standardized mortality ratio"[Abstract] OR "case fatality"[Abstract] OR "relative risk of death"[Abstract] OR "excess mortality"[Abstract] OR "survival"[Abstract]) NOT (animals[MeSH] NOT humans[MeSH])

The dates of the search were 01/01/1980–2/5/2021. 7106 hits were returned, of which 65 were extracted (see PRISMA diagram below) using a structured template to collect information by study for mean and uncertainty for the disease parameter of interest and metadata on age, sex, location, and year. We excluded literature that was not representative of the general population or included pulmonary hypertension Groups 2-5.

Our review of the literature yielded studies from 44 locations, including: Argentina, Australia, Australasia, Belgium, Brazil, Canada, Czechia, Denmark, Finland, France, Germany, India, Ireland, Israel, Japan[Chiba, Miyagi], Mexico, Netherlands, New Zealand, Portugal, Republic of Korea, Russian Federation, Saudi Arabia, Scotland, Singapore, Spain, Sub-Saharan Africa, Sweden, Switzerland, Taiwan (Province of China), United Kingdom [Greater London, Sheffield], United States of America [California, Colorado, Florida, Illinois, Minnesota, New Mexico, New York, Ohio, Pennsylvania]. Years of included studies ranged from 1977 to 2016; more than 75% of the data sources were from 2000 or later.

Source counts

Measure	Total sources	Countries with data
Prevalence	11	9
Incidence	14	11
With-condition mortality rate	55	26

In 70% of the reference studies, RHC was used to confirm the diagnosis of pulmonary arterial hypertension. For 18% of reference studies, a combination of RHC and echocardiography were used; however, an explicit breakdown of the proportion of each modality was not provided. For 8% of reference studies, the primary classification was based in ICD codes; however, medical record review and confirmation was specifically noted. Only 4% of studies used echocardiography alone to establish the diagnosis of PAH.

We did not incorporate cause-specific mortality estimates (CSMR) from death certificates as estimates of survival or case fatality were commonly found in the literature and were measured with a higher degree of precision and alignment with the GBD case definitions than could be determined for death certificates. Due to evolving ICD codes and PAH coding practices on death certificates, we decided published estimates of survival from cohort and other population-based studies of patients with PAH would more closely approximate non-fatal patterns than CSMR from death certificates.

Figure 2: PRISMA 2020 Flow Diagram

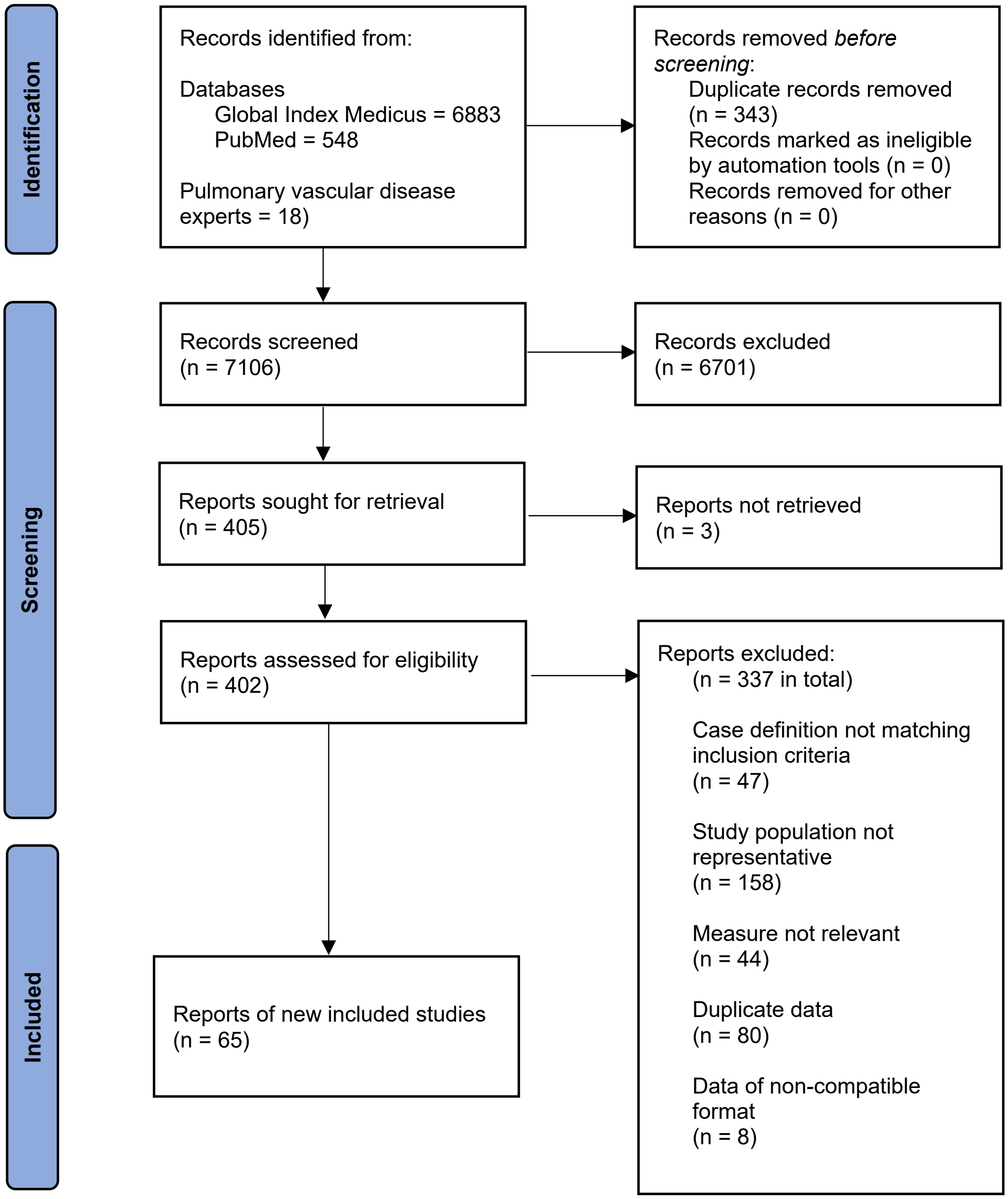

Data processing

We used the modelling software meta-regression—Bayesian, regularized, trimmed (MR-BRT) to split both-sex datapoints for incidence, prevalence, and with-condition mortality into sex-specific estimates. This methodology is detailed elsewhere in the appendix. We also split datapoints where the age range was greater than 25 years. Age splitting was based on the global sex-specific age pattern from a DisMod-MR 2.1 model that only used input data from scientific literature with less than a 25-year age range.

We relied on published estimates of PAH survival or case fatality, transformed from case fatality into with-condition mortality rate using the following formula:

$$mtwith = -\ln(1 - cfr)/time(years)$$

Severity Distributions

Severity distributions, details on the health states for pulmonary arterial hypertension in GBD 2021, and the associated disability weight (DW) are shown in Table 1. We selected heart failure disability weights as most closely representing the disability due to PAH, based on lay descriptions of the health states.

Table 1. Severity distributions and associated disability weights (DW)

Severity level	Lay description	DW (95% CI)
Controlled, medically managed	Has been diagnosed with clinical heart failure, a chronic disease that requires medication every day and causes some worry but minimal interference with daily activities.	0.049 (0.031–0.072)
Mild	Is short of breath and easily tires with moderate physical activity, such as walking uphill or more than a quarter-mile on level ground. The person feels comfortable at rest or during activities requiring less effort.	0.041 (0.026–0.062)
Moderate	Is short of breath and easily tires with minimal physical activity, such as walking only a short distance. The person feels comfortable at rest but avoids moderate activity.	0.072 (0.047–0.103)
Severe	Is short of breath and feels tired when at rest. The person avoids any physical activity, for fear of worsening the breathing problems.	0.179 (0.122–0.251)

Modelling strategy

We used DisMod to model the incidence and prevalence of PAH, informed by the input data described above. We set a prior of no remission and used the Healthcare Access and Quality (HAQ) Index, the natural log of age-standardised schistosomiasis prevalence, and an age-standardised summary exposure value (SEV) scalar for HIV prevalence as covariates. HIV and schistosomiasis were chosen as covariates because these diseases can cause PAH and are drivers of PAH prevalence in locations where those diseases are common. Information on covariates, including parameters and coefficients can be found in Table 2. All data adjustments were done outside of DisMod, described above.

Table 2. Summary of covariates used in the PAH DisMod meta-regression model.

Covariate	Type	Parameter	Exponentiated beta (95% uncertainty interval)
Log-transformed age-standardised SEV scalar: HIV	Country-level	Prevalence	0.45 (0.13 to 0.78)
Log-transformed age-standardised prevalence of schistosomiasis	Country-level	Prevalence	5.64 (1.52 to 17.74)
Healthcare Access and Quality Index	Country-level	Excess mortality rate	-1.01 (-1.95 to -0.096)

Estimates for pulmonary arterial hypertension are being reported for the first time in GBD 2021. As such, there have been no changes from prior rounds.

1 Naghavi M, Ong KL, Aali A, *et al.* Global burden of 288 causes of death and life expectancy decomposition in 204 countries and territories and 811 subnational locations, 1990–2021: a systematic analysis for the Global Burden of Disease Study 2021. *The Lancet* 2024; **403**: 2100–32.

2 Ferrari AJ, Santomauro DF, Aali A, *et al.* Global incidence, prevalence, years lived with disability (YLDs), disability-adjusted life-years (DALYs), and healthy life expectancy (HALE) for 371 diseases and injuries in 204 countries and territories and 811 subnational locations, 1990–2021: a systematic analysis for the Global Burden of Disease Study 2021. *The Lancet* 2024; **403**: 2133–61.

3 Foreman KJ, Lozano R, Lopez AD, Murray CJ. Modeling causes of death: an integrated approach using CODEm. *Popul Health Metr* 2012; **10**: 1.

4 Bell RM, Koren Y. Lessons from the Netflix prize challenge. *SIGKDD Explor Newsl* 2007; **9**: 75–9.

5 Bell RM, Koren Y, Volinsky C. All Together Now: A Perspective on the Netflix Prize. *CHANCE* 2010; **23**: 24–9.

6 Vasudevan S, Ramos F, Nettleton E, Durrant-Whyte H, Blair A. Gaussian Process modeling of large scale terrain. In: 2009 IEEE International Conference on Robotics and Automation. Kobe: IEEE, 2009: 1047–53.

7 Rasmussen CE, Williams CKI. Gaussian Processes for Machine Learning. The MIT Press, 2005 DOI:10.7551/mitpress/3206.001.0001.

8 Murray CJ, Lopez AD. Global mortality, disability, and the contribution of risk factors: Global Burden of Disease Study. *The Lancet* 1997; **349**: 1436–42.

9 Ng M, Freeman MK, Fleming TD, *et al.* Smoking prevalence and cigarette consumption in 187 countries, 1980-2012. *JAMA* 2014; **311**: 183–92.

10 Ng M, Fleming T, Robinson M, *et al.* Global, regional and national prevalence of overweight and obesity in children and adults 1980-2013: A systematic analysis. *Lancet* 2014; **384**: 766–81.

11 Flaxman AD, Vos T, Murray CJL, Kiyono P, editors. An integrative metaregression framework for descriptive epidemiology, 1 edition. Seattle: University of Washington Press, 2015.

12 Link J, Glazer C, Torres F, Chin K. International Classification of Diseases Coding Changes Lead to Profound Declines in Reported Idiopathic Pulmonary Arterial Hypertension Mortality and Hospitalizations. *Chest* 2011; **139**: 497–504.

13 George MG, Schieb LJ, Ayala C, Talwalkar A, Levant S. Pulmonary Hypertension Surveillance. *Chest* 2014; **146**: 476–95.

14 Simonneau G, Robbins IM, Beghetti M, *et al.* Updated Clinical Classification of Pulmonary Hypertension. *Journal of the American College of Cardiology* 2009; **54**: S43–54.
