# Supplementary material for: Global, regional, and national burden of pulmonary arterial hypertension, 1990–2021: a systematic analysis for the Global Burden of Disease Study 2021
Source: Lancet Respir Med. 2025 Jan;13(1):69–79. doi: 10.1016/S2213-2600(24)00295-9 (PMC11698691; doi:10.1016/S2213-2600(24)00295-9)
Supplement: Supplementary appendix 2 [file mmc2.pdf]

THE LANCET
Respiratory Medicine

Supplementary appendix 2

This appendix formed part of the original submission and has been peer reviewed. We post it as supplied by the authors.

Supplement to: GBD 2021 Pulmonary Arterial Hypertension Collaborators. Global, regional, and national burden of pulmonary arterial hypertension, 1990–2021: a systematic analysis for the Global Burden of Disease Study 2021. *Lancet Respir Med* 2024; published online Oct 18. https://doi.org/10.1016/S2213-2600(24)00295-9.

Appendix 2: Authorship appendix to "Global, Regional and National Burden of Pulmonary Arterial Hypertension, 1990-2021: a systematic analysis for the Global Burden of Disease Study 2021"

This appendix provides further authorship detail for "Global, Regional and National Burden of Pulmonary Arterial Hypertension, 1990-2021: a systematic analysis for the Global Burden of Disease Study 2021."

Table of Contents

GBD 2021 Pulmonary Arterial Hypertension Collaborators 2

Affiliations 3

Authors' Contributions 10

- Managing the overall research enterprise 10
- Writing the first draft of the manuscript 10
- Primary responsibility for applying analytical methods to produce estimates 11
- Primary responsibility for seeking, cataloguing, extracting, or cleaning data; designing or coding figures and tables 11
- Providing data or critical feedback on data sources 11
- Developing methods or computational machinery 11
- Providing critical feedback on methods or results 12
- Drafting the work or revising it critically for important intellectual content 13
- Managing the estimation or publications process 14

GBD 2021 Pulmonary Arterial Hypertension Collaborators

Peter J Leary, Megan Lindstrom, Catherine O Johnson, Sophia Emmons-Bell, Stuart Rich, Paul A Corris, Hilary M DuBrock, Corey E Ventetuolo, Yohannes Habtegiorgis Abate, Michael Abdelmasseh, Richard Gyan Aboagye, Hasan Abualruz, Eman Abu-Gharbieh, Salahdein Aburuz, Lawan Hassan Adamu, Rui Adão, Isaac Yeboah Addo, Rufus Adesoji Adedoyin, Juliana Bunmi Adetunji, Leticia Akua Adzigbli, Bright Opoku Ahinkorah, Firdos Ahmad, Amir Mahmoud Ahmadzade, Ayman Ahmed, Haroon Ahmed, Syed Anees Ahmed, Shiva Akhlaghi, Mohammed Ahmed Akkaif, Salah Al Awaidy, Samer O Alalalmeh, Almaza Albakri, Khalifah A Aldawsari, Wael Almahmeed, Najim Z Alshahrani, Awais Altaf, Hany Aly, Karem H Alzoubi, Walid Adnan Al-Zyoud, Reza Amani, Ganiyu Adeniyi Amusa, Catalina Liliana Andrei, Saleha Anwar, Anayochukwu Edward Anyasodor, Aleksandr Y Aravkin, Demelash Areda, Haftu Asmerom Asmerom, Avinash Aujayeb, Ahmed Y. Azzam, Abraham Samuel Babu, Sara Bagherieh, Ovidiu Constantin Baltatu, Hiba Jawdat Barqawi, Mohammad-Mahdi Bastan, Kavita Batra, Nebiyou Simegnew Bayleyegn, Amir Hossein Behnoush, Jaideep Singh Bhalla, Sonu Bhaskar, Vivek Bhat, Saeid Bitaraf, Veera R Bitra, Archith Boloor, Dejana Braithwaite, Michael Brauer, Lemma N Bulto, Yasser Bustanji, Vijay Kumar Chattu, Gerald Chi, Fatemeh Chichagi, Bryan Chong, Rajiv Chowdhury, Zinhle Cindi, Natalia Cruz-Martins, Sriharsha Dadana, Omid Dadras, Tukur Dahiru, Xiaochen Dai, Mohadese Dashtkoohi, Sean DeAngelo, Shayom Debopadhaya, Berecha Hundessa Demessa, Hardik Dineshbhai Desai, Vishal R Dhulipala, Michael J Diaz, Mengistie Diress, Thanh Chi Do, Thao Huynh Phuong Do, Khanh Duy Doan, Wendel Mombaque dos Santos, Rajkumar Prakashbhai Doshi, Robert Kokou Dowou, Arkadiusz Marian Dziedzic, Muhammed Elhadi, Farshid Etaee, Natalia Fabin, Adeniyi Francis Fagbamigbe, Pawan Sirwan Faris, Bikila Regassa Feyisa, Celia Fortuna Rodrigues, Aravind P Gandhi, Mohammad Arfat Ganiyani, Yibeltal Yismaw Gela, Molla Getie, Amir Ghaffari Jolfayi, Afsaneh Ghasemzadeh, Mohamad Goldust, Mahaveer Golechha, Shi-Yang Guan, Mesay Dechasa Gudeta, Mohak Gupta, Rahul Gupta, Mostafa Hadei, Ahmad Hammoud, Md Saquib Hasnain, Mahgol Sadat Hassan Zadeh Tabatabaei, Simon I Hay, Omar E Hegazi, Mehdi Hemmati, Yuta Hiraike, Nguyen Quoc Hoan, Michael Hultström, Hong-Han Huynh, Segun Emmanuel Ibitoye, Olayinka Stephen Ilesanmi, Nahlah Elkudssiah Ismail, Chidozie Declan Iwu, Khushleen Jaggi, Akhil Jain, Mihajlo Jakovljevic, Sun Ha Jee, Bijay Mukesh Jeswani, Anil K Jha, Mohammad Jokar, Nitin Joseph, Jacek Jerzy Jozwiak, Hannaneh Kabir, Farima Kahe, Arun Kamireddy, Arun R Kanmanthareddy, Hanie Karimi, Arman Karimi Behnagh, Sina Kazemian, Pedram Keshavarz, Amirmohammad Khalaji, Mohammad Jobair Khan, Feriha Fatima Khidri, Min Seo Kim, Shivakumar KM Marulasiddaiah Kondlahalli, Nikhil Kothari, Kewal Krishan, Mukhtar Kulimbet, Ashish Kumar, Kaveh Latifinaibin, Thao Thi Thu Le, Caterina Ledda, Seung Won Lee, Ming-Chieh Li, Stephen S Lim, Shuke Liu, Elham Mahmoudi, Omar M Makram, Kashish Malhotra, Ahmad Azam Malik, Deborah Carvalho Malta, Yosef Manla, Miquel Martorell, Kamran Mehrabani-Zeinabad, Mohsen Merati, Tomislav Mestrovic, Niloofar Mirdamadi, Arup Kumar Misra, Ali H Mokdad, Mohammad Ali Moni, AmirAli Moodi Ghalibaf, Paula Moraga, Negar Morovatdar, Rohith Motappa, Seyed Ali Mousavi-Aghdas, Ahmad Mustafa, Ganesh R Naik, Mohammad Sadeq Najafi, Soroush Najdaghi, Dhairya P Nanavaty, Delaram Narimani Davani, Zuhair S Natto, Javaid Nauman, Dang H Nguyen, Phat Tuan Nguyen, Robina Khan Niazi, Bogdan Oancea, Titilope O Olanipekun, Gláucia Maria Moraes Oliveira, Hany A Omar, Mahesh Padukudru P A, Feng Pan, Seithikurippu R Pandi-Perumal, Ioannis Pantazopoulos, Romil R Parikh, Ionela-Roxana Petcu, Hoang Nhat Pham, Hoang Tran Pham, Anil K Philip, Elton Junio Sady Prates, Jagadeesh Puvvula, Gangzhen Qian, Quinn Rafferty, Fakher Rahim, Mehran Rahimi, Mosiur Rahman, Muhammad Aziz Rahman, Mohammad Rahmanian, Nazanin Rahmanian, Masoud Rahmati, Rahem Rahmati, Mahmoud Mohammed Ramadan, Kamleshun Ramphul, Juwel Rana, Indu Ramachandra Rao, Sina Rashedi, Nakul Ravikumar, Salman Rawaf, Ayita Ray, Murali

Mohan Rama Krishna Reddy, Elrashdy Moustafa Mohamed Redwan, Negar Rezaei, Priyanka Roy, Aly M A Saad, Basema Ahmad Saddik, Masoumeh Sadeghi, Mohammad Reza Saeb, Fatemeh Saheb Sharif-Askari, Narjes Saheb Sharif-Askari, Mohamed A Saleh, Najib Yahaya Sani, Ushasi Saraswati, Aswini Saravanan, Jennifer Saulam, Art Schuermans, Austin E Schumacher, Birhan Ewunu Semagn, Yashendra Sethi, Allen Seylani, Melika Shafeghat, Moyad Jamal Shahwan, Muhammad Aaqib Shamim, Anas Shamsi, Sadaf Sharfaei, Kamal Sharma, Nitish Sharma, Akil Adrian Sherif, Ivy Shiue, Seyed Afshin Shorofi, Emmanuel Edwar Siddig, Harpreet Singh, Jasvinder A Singh, Paramdeep Singh, Surjit Singh, Farrukh Sobia, Ranjan Solanki, Shipra Solanki, Michael Spartalis, Chandan Kumar Swain, Lukasz Szarpak, Seyyed Mohammad Tabatabaei, Celine Tabche, Jacques Lukenze Tamuzi, Ker-Kan Tan, Masayuki Teramoto, Samar Tharwat, Friedrich Thienemann, Thien Tan Tri Tai Truyen, Guesh Mebrahtom Tsegay, Aniefiok John Udoakang, Jef Van den Eynde, Shoban Babu Varthya, Madhur Verma, Dominique Vervoort, Manish Vinayak, Maria Viskadourou, Fang Wang, Nuwan Darshana Wickramasinghe, Angga Wilandika, Suowen Xu, Chuanhua Yu, Iman Zare, Mohammad A Zeineddine, Zhi-Jiang Zhang, Lei Zhu, Abzal Zhumagaliuly, Magdalena Zielińska, Samer H Zyoud, Christopher J L Murray, and Gregory A Roth.

Affiliations

School of Medicine (P J Leary PhD), Institute for Health Metrics and Evaluation (M Lindstrom PhD, C O Johnson PhD, A Y Aravkin PhD, Prof M Brauer DSc, X Dai PhD, Prof S I Hay FMedSci, Prof S S Lim PhD, T Mestrovic PhD, Prof A H Mokdad PhD, Q Rafferty BA, A E Schumacher PhD, Prof C J L Murray DPhil, G A Roth MD), Department of Applied Mathematics (A Y Aravkin PhD), Department of Health Metrics Sciences, School of Medicine (A Y Aravkin PhD, X Dai PhD, Prof S I Hay FMedSci, Prof S S Lim PhD, Prof A H Mokdad PhD, Prof C J L Murray DPhil, G A Roth MD), School of Health Systems and Public Health (C Iwu MPH), Division of Cardiology (G A Roth MD), University of Washington, Seattle, WA, USA; Department of Global Health (S Emmons-Bell MPH), Seattle, WA, USA; Department of Medicine (Prof S Rich MD), Northwestern University, Chicago, IL, USA; Institute of Translational and Clinical Research (Prof P A Corris MD), Newcastle University, Newcastle Upon Tyne, UK; Pulmonary Vascular Research Institute, London, UK (Prof P A Corris MD); Department of Internal Medicine (H M DuBrock MD), Department of Cardiovascular Medicine (H Pham MD), Mayo Clinic, Rochester, MN, USA; Department of Medicine (C E Ventetuolo MD), Brown University, Providence, RI, USA; Department of Medicine (C E Ventetuolo MD), Rhode Island Hospital, Providence, RI, USA; Department of Clinical Governance and Quality Improvement (Y H Abate MSc), Aleta Wondo General Hospital, Aleta Wondo, Ethiopia; Department of Surgery (M Abdelmasseh MD), Marshall University, Huntington, WV, USA; Department of Family and Community Health (R G Aboagye MPH), Department of Epidemiology and Biostatistics (L A Adzigbli BSc, R K Dowou MPhil), University of Health and Allied Sciences, Ho, Ghana; Department of Nursing (H Abualruz PhD), Al Zaytoonah University of Jordan, Amman, Jordan; Department of Biopharmaceutics and Clinical Pharmacy (Prof E Abu-Gharbieh PhD), School of Pharmacy (Prof Y Bustanji PhD), The University of Jordan, Amman, Jordan; Clinical Sciences Department (Prof E Abu-Gharbieh PhD, H J Barqawi MPhil, N Saheb Sharif-Askari PhD), College of Medicine (F Ahmad PhD, Prof B A Saddik PhD, Prof M A Saleh PhD), Department of Pharmacy Practice and Pharmacotherapeutics (Prof K H Alzoubi PhD, Prof H A Omar PhD), Department of Basic Biomedical Sciences (Prof Y Bustanji PhD), Department of Clinical Science (Prof M M Ramadan PhD), Sharjah Institute of Medical Sciences (F Saheb Sharif-Askari PhD), University of Sharjah, Sharjah, United Arab Emirates; Department of Therapeutics (Prof S Aburuz PhD), College of Medicine and Health Sciences (J Nauman PhD), United Arab Emirates University, Al Ain, United Arab Emirates; College of Pharmacy (Prof S Aburuz PhD), University of Jordan, Amman, Jordan; Department of Human Anatomy (L H Adamu PhD), Federal University Dutse, Dutse, Nigeria; Department

of Anatomy (L H Adamu PhD), Bayero University Kano, Kano, Nigeria; Department of Pharmacology and Toxicology (Prof R Adão PhD), Complutense University of Madrid, Madrid, Spain; Department of Surgery and Physiology (Prof R Adão PhD), Institute for Research and Innovation in Health (i3S) (Prof N Cruz-Martins PhD), Associate Laboratory Institute for Health and Bioeconomy (i4HB) (Prof C Fortuna Rodrigues PhD), Faculty of Engineering (Prof C Fortuna Rodrigues PhD), University of Porto, Porto, Portugal; School of Medicine (I Y Addo PhD), University of Sydney, Sydney, NSW, Australia; Centre for Social Research in Health (I Y Addo PhD), School of Population Health (Prof B A Saddik PhD), University of New South Wales, Sydney, NSW, Australia; Department of Medical Rehabilitation (Prof R A Adedoyin PhD), Obafemi Awolowo University, Ile-Ife, Nigeria; Department of Biochemistry (J B Adetunji PhD), Osun State University, Osogbo, Nigeria; School of Public Health (B O Ahinkorah MPhil), University of Technology Sydney, Sydney, NSW, Australia; Department of Neuroscience (A Ahmadzade MD), Clinical Research Development Unit (N Morovatdar MD), Department of Medical Informatics (S Tabatabaei PhD), Clinial Research Development Unit (S Tabatabaei PhD), Mashhad University of Medical Sciences, Mashhad, Iran; Institute of Endemic Diseases (A Ahmed MSc), Unit of Basic Medical Sciences (E E Siddig MD), University of Khartoum, Khartoum, Sudan; Swiss Tropical and Public Health Institute (A Ahmed MSc), University of Basel, Basel, Switzerland; Department of Biosciences (H Ahmed PhD), COMSATS Institute of Information Technology, Islamabad, Pakistan; Brody School of Medicine (S Ahmed PhD), East Carolina University, Greenville, NC, USA; Department of Medicinal Chemistry (S Akhlaghi MSc), Department of Community Medicine (Prof S Bitaraf PhD), Ahvaz Jundishapur University of Medical Sciences, Ahvaz, Iran; Department of Cardiology (M Akkaif PhD), Fudan University, Shanghai, China; Department of Communicable Diseases (S Al Awaidy MSc), Ministry of Health, Muscat, Oman; Middle East, Eurasia, and Africa Influenza Stakeholders Network, Muscat, Oman (S Al Awaidy MSc); Department of Clinical Sciences (S O Alalalmeh BPharm, O E Hegazi BPharm), Center for Medical and Bio-Allied Health Sciences Research (Prof M J Shahwan PhD, A Shamsi PhD, S H Zyoud PhD), Ajman University, Ajman, United Arab Emirates; Department of Medicine (A Albakri MD), Royal Jordanian Medical Services, Amman, Jordan; Department of Pediatrics (K A Aldawsari MD), Nicklaus Children's Hospital, Miami, FL, USA; Heart Center (K A Aldawsari MD), King Faisal Specialist Hospital & Research Center, Riyadh, Saudi Arabia; Department of Cardiology, Heart, Vascular, and Thoracic Institute (Prof W Almahmeed MD), Cleveland Clinic Abu Dhabi, Abu Dhabi, United Arab Emirates; College of Medicine and Health Sciences Academic Programs (Prof W Almahmeed MD), Khalifa University, Abu Dhabi, United Arab Emirates; Department of Family and Community Medicine (N Z Alshahrani MD), University of Jeddah, Jeddah, Saudi Arabia; Institute of Molecular Biology and Biotechnology (A Altaf PhD), The University of Lahore, Lahore, Pakistan; Department of Pediatrics (Prof H Aly MD), Department of Internal Medicine (J Bhalla MD, M Gupta MD, U Saraswati MD), Cleveland Clinic, Cleveland, OH, USA; Department of Clinical Pharmacy (Prof K H Alzoubi PhD), Jordan University of Science and Technology, Irbid, Jordan; Department of Biomedical Engineering (W A Al-Zyoud PhD), German Jordanian University, Amman, Jordan; Interdisciplinary Graduate Program in Human Toxicology (R Amani DVM), University of Iowa, Iowa City, IA, USA; Health Policy Research Center (R Amani DVM), Shiraz University of Medical Sciences, Shiraz, Iran; Department of Medicine (G A Amusa MD), University of Jos, Jos, Nigeria; Department of Internal Medicine (G A Amusa MD), Jos University Teaching Hospital, Jos, Nigeria; Department of Cardiology (Prof C Andrei PhD), Carol Davila University of Medicine and Pharmacy, Bucharest, Romania; Centre for Interdisciplinary Research in Basic Sciences (CIRBSc) (S Anwar PhD), Centre For Interdisciplinary Research In Basic Sciences (CIRBSc) (A Shamsi PhD), Jamia Millia Islamia, New Delhi, India; School of Chemical and Life Sciences (SCLS) (S Anwar PhD), Jamia Hamdard, New Delhi,

India; Rural Health Research Institute (A E Anyasodor PhD), Charles Sturt University, Orange, NSW, Australia; College of Art and Science (D Areda PhD), Ottawa University, Surprise, AZ, USA; School of Life Sciences (D Areda PhD), Arizona State University, Tempe, AZ, USA; School of Medical Laboratory Sciences (H A Asmerom MSc), Department of Clinical Pharmacy (M D Gudeta MSc), Haramaya University, Harar, Ethiopia; Northumbria HealthCare NHS Foundation Trust, Newcastle upon Tyne, UK (A Aujayeb MBBS); Montefiore-Einstein Cerebrovascular Research Lab (A Azzam MBBCh), Albert Einstein College of Medicine, Bronx, NY, USA; Faculty of Medicine (A Azzam MBBCh), Department of Cardiology (O M Makram MD), October 6 University, 6th of October City, Egypt; Department of Physiotherapy (A S Babu PhD), Department of Nephrology (I Rao DM), Manipal Academy of Higher Education, Manipal, India; School of Medicine (S Bagherieh BSc), Department of Epidemiology and Biostatistics (K Mehrabani-Zeinabad PhD), Heart Failure Research Center (S Najdaghi MD, D Narimani Davani MD), Neuroscience Research Center (S Najdaghi MD), Cardiac Rehabilitation Research Center (Prof M Sadeghi MD), Isfahan University of Medical Sciences, Isfahan, Iran; College of Medicine (Prof O Baltatu PhD), Alfaisal University, Riyadh, Saudi Arabia; Center of Innovation, Technology and Education (CITE) (Prof O Baltatu PhD), Anhembi Morumbi University, São José dos Campos, Brazil; Non-communicable Diseases Research Center (M Bastan MD, N Rezaei PhD), School of Medicine (A Behnoush BS, H Karimi MD, A Khalaji MD, M Merati MD, M Shafeghat MD), Department of Scientific Research (F Chichagi MD), Iranian Research Center for HIV/AIDS (IRCHA) (O Dadras PhD), Department of Obstetrics and Gynecology (M Dashtkoohi MD), Department of Health in Emergencies and Disasters (M Hadei PhD), Sina Trauma and Surgery Research Center (M Hassan Zadeh Tabatabaei MD), Cardiac Primary Prevention Research Center (S Kazemian MD), Department of Cardiac Electrophysiology (S Kazemian MD), Department of Cardiology (E Mahmoudi MD), Endocrinology and Metabolism Research Institute (N Mirdamadi MD, N Rezaei PhD), Sports and Exercise Medicine Research Center (N Mirdamadi MD), Tehran Heart Center (M Najafi MD), Research Center for Advanced Technologies in Cardiovascular Medicine (M Najafi MD), Tehran University of Medical Sciences, Tehran, Iran; School of Medicine (M Bastan MD), Department of Cardiology (A Ghaffari Jolfayi MD), Endocrine Research Center (A Karimi Behnagh MD), Department of Echocardiography (A Karimi Behnagh MD), Department of Anesthesiology (K Latifinaibin MD), Iran University of Medical Sciences, Tehran, Iran; Department of Medical Education (K Batra PhD), University of Nevada Las Vegas, Las Vegas, NV, USA; Department of Surgery (N S Bayleyegn MD), Jimma University, Jimma, Ethiopia (B Feyisa MPH); Endocrinology and Metabolism Research Institute (A Khalaji MD), Department of Epidemiology (S Rashedi MD), Non-Communicable Diseases Research Center (NCDRC), Tehran, Iran (A Behnoush BS); Department of Cardiovascular Medicine (J Bhalla MD, U Saraswati MD), Mayo Clinic Foundation for Medical Education and Research, Rochester, MN, USA; Global Health Neurology Lab (S Bhaskar PhD), NSW Brain Clot Bank, Sydney, NSW, Australia; Division of Cerebrovascular Medicine and Neurology (S Bhaskar PhD), National Cerebral and Cardiovascular Center, Suita, Japan; Department of Internal Medicine (V Bhat MBBS), St. John's National Academy of Health Sciences, Bangalore, India; Faculty of Health Sciences (V R Bitra PhD), University of Botswana, Gaborone, Botswana; Department of Internal Medicine (A Boloor MD, M M R Reddy MD), Department of Community Medicine (N Joseph MD, R Motappa MD), Manipal Academy of Higher Education, Mangalore, India; Department of Epidemiology (D Braithwaite PhD), College of Medicine (M J Diaz BS), University of Florida, Gainesville, FL, USA; Cancer Population Sciences Program (D Braithwaite PhD), University of Florida Health Cancer Center, Gainesville, FL, USA; School of Population and Public Health (Prof M Brauer DSc), University of British Columbia, Vancouver, BC, Canada; College of Nursing and Health Sciences (L N Bulto PhD), College of Medicine and Public Health (G R Naik PhD), Department of

Nursing and Health Sciences (S Shorofi PhD), Flinders University, Adelaide, SA, Australia; Temerty Faculty of Medicine (V Chattu MD), University of Toronto, Toronto, ON, Canada; Department of Community Medicine (V Chattu MD), Datta Meghe Institute of Medical Sciences, Sawangi, India; Division of Cardiovascular Medicine (G Chi MD), Department of Health Policy and Oral Epidemiology (Z S Natto DrPH), Department of Pulmonary and Critical Care (T O Olanipekun MD), Beth Israel Deaconess Medical Center (S Sharfaei MD), Harvard University, Boston, MA, USA; Department of Medicine (B Chong MBBS), Department of Surgery (K Tan PhD), National University of Singapore, Singapore, Singapore; Florida International University, Miami, FL, USA (Prof R Chowdhury PhD); Department of Epidemiology (Prof R Chowdhury PhD), Department of Emergency Medicine (Prof I Pantazopoulos PhD), University of Bern, Bern, Switzerland; Department of Genetics (Z Cindi PhD), Department of Biostatistics, Epidemiology, and Informatics (J Puvvula PhD), University of Pennsylvania, Philadelphia, PA, USA; Department of Diagnostic and Therapeutic Technologies (Prof N Cruz-Martins PhD), Cooperativa de Ensino Superior Politécnico e Universitário (Polytechnic and University Higher Education Cooperative), Vila Nova de Famalicão, Portugal; Department of Internal Medicine (S Dadana MD), Cheyenne Regional Medical Center, Cheyenne, WY, USA; Department of Global Public Health and Primary Care (O Dadras PhD), University of Bergen, Bergen, Norway; Department of Community Medicine (Prof T Dahiru MA), Ahmadu Bello University, Zaria, Nigeria; Department of Gynecology and Obstetrics (M Dashtkoohi MD), Vali-E-Asr Reproductive Health Research Center, Tehran, Iran; Department of Internal Medicine (S DeAngelo DO), John H. Stroger, Jr. Hospital of Cook County, Chicago, IL, USA; Medical College (S Debopadhaya BS), Albany Medical College, Albany, NY, USA; USAID-JSI Digital Health Activity (B H Demessa MPH), Jimma University, Addis Ababa, Ethiopia; Department of Research (H D Desai MD), Gujarat Adani Institute of Medical Sciences and G.K. General Hospital, Bhuj, India; The Zena and Michael A. Wiener Cardiovascular Institute (V R Dhulipala MD), Department of Cardiology (M Vinayak MD), Icahn School of Medicine at Mount Sinai, New York, NY, USA; Department of Human Physiology (M Diress MSc, Y Gela MSc), University of Gondar, Gondar, Ethiopia; Department of Medicine (T C Do MD), Pham Ngoc Thach University of Medicine, Ho Chi Minh City, Vietnam; Department of Medicine (T H Do MD), Can Tho University of Medicine and Pharmacy, Can Tho, Vietnam; Department of Pathology (K Doan MD), Temple University Hospital, Philadelphia, PA, USA; Departamento de Responsabilidade Social (Department of Social Responsibility) (W M dos Santos PhD), Oswaldo Cruz German Hospital, São Paulo, Brazil; Brazilian Centre for Evidence-based Healthcare (W M dos Santos PhD), Joanna Briggs Institute, São Paulo, Brazil; Department of Cardiology (R P Doshi MD), Hackettstown Medical Center, Hackettstown, NJ, USA; Newton Medical Center, Sparta, NJ, USA (R P Doshi MD); Department of Conservative Dentistry with Endodontics (A M Dziedzic DSc), Medical University of Silesia, Katowice, Poland; Faculty of Medicine (M Elhadi MD), University of Tripoli, Tripoli, Libya; Houston Methodist Hospital, Houston, TX, USA (M Elhadi MD); Department of Internal Medicine (F Etaee MD), Department of Dermatology (M Goldust MD), Yale University, New Haven, CT, USA; Independent Consultant, Bologna, Italy (N Fabin MD); Department of Epidemiology and Medical Statistics (A F Fagbamigbe PhD), Department of Health Promotion and Education (S Ibitoye PhD), University of Ibadan, Ibadan, Nigeria; Research Centre for Healthcare and Community (A F Fagbamigbe PhD), Coventry University, Coventry, UK; Department of Biology (P S Faris PhD), Salahaddin University-Erbil, Erbil, Iraq; Department of Biology (P S Faris PhD), Cihan University-Erbil, Erbil, Iraq; Institute of Health Sciences (B Feyisa MPH), Wollega University, Nekemte, Ethiopia; Department of Community Medicine and Family Medicine (A P Gandhi MD), All India Institute of Medical Sciences, Nagpur, India; Department of General Medicine (M Ganiyani MD), Grant Medical College & Sir J.J. Group of Hospitals, Mumbai, India; Department of Medicine (M

Ganiyani MD), Miami Cancer Institute, Miami, FL, USA; Department of Medical Laboratory Science (M Getie MSc), Addis Ababa University, Addis Ababa, Ethiopia; Faculty of Medicine (A Ghaffari Jolfayi MD), Student Research Committee (M Rahmanian MD), Shahid Beheshti University of Medical Sciences, Tehran, Iran; Immunology Research Center (A Ghasemzadeh MD), Tuberculosis and Lung Diseases Research Center (S Mousavi-Aghdas MD), Cardiovascular Research Center (M Rahimi MD), Tabriz University of Medical Sciences, Tabriz, Iran; Department of Health Systems and Policy Research (M Golechha PhD), Indian Institute of Public Health, Gandhinagar, India; Department of Epidemiology and Biostatistics (S Guan MD), Anhui Medical University, Hefei, China; Department of Cardiology (R Gupta MBBS), Lehigh Valley Health Network, Allentown, PA, USA; Department of Medical and Technical Information Technology (A Hammoud PhD), Bauman Moscow State Technical University, Moscow, Russia; Department of Pharmacy (Prof M S Hasnain PhD), Palamau Institute of Pharmacy, Daltonganj, India; Department of Medicine (M Hemmati MD), MedStar Health, Washington, DC, USA; Department of Medicine (M Hemmati MD), Georgetown University, Washington DC, DC, USA; Division for Health Service Promotion (Y Hiraike PhD), University of Tokyo, Tokyo, Japan; School of Dentistry (N Hoan DDS), Hanoi Medical University, Hanoi, Vietnam; Department of Surgical Sciences (M Hultström PhD), Department of Medical Cell Biology (M Hultström PhD), Uppsala University, Uppsala, Sweden; International Master Program for Translational Science (H Huynh BS), Taipei Medical University, Taipei, Taiwan; West Africa RCC (O S Ilesanmi PhD), Africa Centre for Disease Control and Prevention, Abuja, Nigeria; Department of Community Medicine (O S Ilesanmi PhD), University College Hospital, Ibadan, Ibadan, Nigeria; Department of Clinical Pharmacy & Pharmacy Practice (Prof N Ismail PhD), Asian Institute of Medicine, Science and Technology, Bedong, Malaysia; Malaysian Academy of Pharmacy, Puchong, Malaysia (Prof N Ismail PhD); Department of Nephrology (K Jaggi MD), San Mateo Medical Center, San Mateo, CA, USA; Department of Nephrology (K Jaggi MD), Mills Peninsula Medical Center, Burlingame, CA, USA; Department of Leukemia (A Jain MD), The University of MD Anderson Cancer Center, Houston, TX, USA; The World Academy of Sciences UNESCO, Trieste, Italy (Prof M Jakovljevic PhD); Shaanxi University of Technology, Hanzhong, China (Prof M Jakovljevic PhD); Department of Epidemiology and Health Promotion (Prof S Jee PhD), Yonsei University, Seoul, South Korea; Department of Internal Medicine (B M Jeswani MBBS), GCS Medical College, Hospital & Research Centre, Ahmedabad, India; Department of Cardiovascular Medicine (A K Jha MD), Interventional Cardiology Department (A Ray MD), Saint Vincent Hospital, Worcester, MA, USA; Faculty of Veterinary Medicine (M Jokar DVM), University of Calgary, Calgary, Canada; Young Researchers and Elite Club (M Jokar DVM), Islamic Azad University, Karaj, Iran; Department of Family Medicine and Public Health (J J Jozwiak PhD), University of Opole, Opole, Poland; Department of Bioengineering (H Kabir MSc), University of California Berkeley, Berkeley, CA, USA; Department of Internal Medicine (F Kahe MD), Wayne State University, Detroit, MI, USA; Russell H. Morgan Department of Radiology and Radiological Science (A Kamireddy MD), Department of Health Policy and Management (D Vervoort MD), Division of Cardiology (M Viskadourou MD), Johns Hopkins University, Baltimore, MD, USA; Cardiology Division (A R Kanmanthareddy MD), Creighton University, Omaha, NE, USA; College of Public Health (A R Kanmanthareddy MD), University of Nebraska Medical Center, Omaha, NE, USA; School of Science and Technology (P Keshavarz MD), The University of Georgia, Tbilisi, Georgia; Department of Diagnostic & Interventional Radiology (P Keshavarz MD), New Hospitals LTD, Tbilisi, Georgia; Department of Rehabilitation Sciences (M Khan MPH), Hong Kong Polytechnic University, Hong Kong, China; Department of Biochemistry (F Khidri PhD), Liaquat University Of Medical and Health Sciences, Jamshoro, Pakistan; Broad Institute of MIT and Harvard, Cambridge, MA, USA (M Kim MD); Division of

Cardiology (D H Nguyen BS), Cardiovascular Research Center (A Schuermans BSc), Massachusetts General Hospital, Boston, MA, USA (M Kim MD); Department of Public Health Dentistry (Prof S M Kondlahalli MD), Krishna Vishwa Vidyapeeth (Deemed to be University), Karad, India; Department of Anaesthesiology and Critical Care (Prof N Kothari PhD), Department of Pharmacology and Research (A Saravanan MD), Department of Pharmacology (M Shamim MBBS, S Singh MD, S B Varthya MD), All India Institute of Medical Sciences, Jodhpur, India; Department of Anthropology (Prof K Krishan PhD), Panjab University, Chandigarh, India; Atchabarov Scientific-Research Institute of Fundamental and Applied Medicine (M Kulimbet MSc, A Zhumagaliuly MD), Kazakh National Medical University, Almaty, Kazakhstan; Center of Medicine and Public Health (M Kulimbet MSc), Asfendiyarov Kazakh National Medical University, Almaty, Kazakhstan; Department of Cardiovascular Medicine (A Kumar MD), Cabrini Institute, Rochester, MN, USA; University of Medicine and Pharmacy at Ho Chi Minh City, Ho Chi Minh City, Vietnam (T T Le MD); Department of Clinical and Experimental Medicine (Prof C Ledda PhD), University of Catania, Catania, Italy; Department of Precision Medicine (Prof S Lee MD), Sungkyunkwan University, Suwon-si, South Korea; Department of Health Promotion and Health Education (M Li PhD), National Taiwan Normal University, Taipei, Taiwan; Department of Cardiology (S Liu MSc, G Qian MS), Guiqian International General Hospital, Guiyang, China; Department of Medicine (O M Makram MD), Medical College of Georgia at Augusta University, Augusta, GA, USA; Rama Medical College Hospital and Research Centre, Uttar Pradesh, India (K Malhotra MBBS); Institute of Applied Health Research (K Malhotra MBBS), University of Birmingham, Birmingham, UK; Rabigh Faculty of Medicine (Prof A Malik PhD), Department of Dental Public Health (Z S Natto DrPH), King Abdulaziz University, Jeddah, Saudi Arabia; Department of Maternal-Child Nursing and Public Health (Prof D C Malta PhD, E J S Prates BS), Federal University of Minas Gerais, Belo Horizonte, Brazil; Smidt Heart Institute (Y Manla MD), Cedars-Sinai Medical Center, Los Angeles, CA, USA; Department of Nutrition and Dietetics (M Martorell PhD), Centre for Healthy Living (M Martorell PhD), University of Concepción, Concepción, Chile; University Centre Varazdin (T Mestrovic PhD), University North, Varazdin, Croatia; Department of Pharmacology (A K Misra MD), All India Institute of Medical Sciences, Mangalagiri, India; AI & Cyber Futures Institute (M Moni PhD), Charles Sturt University, Bathurst, NSW, Australia; The University of Queensland, Brisbane, QLD, Australia (M Moni PhD); Faculty of Medicine (A Moodi Ghalibaf MD), Birjand University of Medical Sciences, Birjand, Iran; Computer, Electrical, and Mathematical Sciences and Engineering Division (P Moraga PhD), King Abdullah University of Science and Technology, Thuwal, Saudi Arabia; Department of Internal Medicine (A Mustafa MD), Staten Island University Hospital Northwell Health, Staten Island, NY, USA; Department of Engineering (G R Naik PhD), Western Sydney University, Sydney, NSW, Australia; Department of Internal Medicine (D P Nanavaty MD), The Brooklyn Hospital Center, Brooklyn, NY, USA; Department of Circulation and Medical Imaging (J Nauman PhD), Norwegian University of Science and Technology, Trondheim, Norway; Department of Medical Engineering (D H Nguyen BS), University of South Florida, Tampa, FL, USA; Department of Surgery (P T Nguyen MD), Danang Family Hospital, Danang, Vietnam; International Islamic University Islamabad, Islamabad, Pakistan (R K Niazi PhD); Department of Applied Economics and Quantitative Analysis (Prof B Oancea PhD), University of Bucharest, Bucharest, Romania; Cardiology Department (G M M Oliveira PhD), Federal University of Rio de Janeiro, Rio de Janeiro, Brazil; Department of Pharmacology and Toxicology (Prof H A Omar PhD), Beni-Suef University, Beni-Suef, Egypt; Department of Respiratory Medicine (Prof M P P A DNB), Jagadguru Sri Shivarathreeswara University, Mysore, India; Menzies Institute for Medical Research (F Pan PhD), University of Tasmania, Hobart, TAS, Australia; Centre for Research and Development (S R Pandi-Perumal MSc), Chandigarh University, Punjab, India; Division of Research and Development (S R

Pandi-Perumal MSc), Lovely Professional University, Phagwara, India; Department of Emergency Medicine (Prof I Pantazopoulos PhD), University of Thessaly, Larissa, Greece; Department of Epidemiology and Community Health (R R Parikh MD), University of Minnesota, Minneapolis, MN, USA; Department of Statistics and Econometrics (I Petcu PhD), Bucharest University of Economic Studies, Bucharest, Romania; Department of Internal Medicine (H Pham MD), University of Arizona, Tucson, AZ, USA; Department of Internal Medicine (H Pham MD), Weiss Memorial Hospital, Chicago, IL, USA; School of Pharmacy (A K Philip PhD), University of Nizwa, Nizwa, Oman; Department of Medical Laboratory Technologies (Prof F Rahim PhD), Al-Noor Center of Research and Innovation (Prof F Rahim PhD), Alnoor University, Mousl, Iraq; Department of Population Science and Human Resource Development (Prof M Rahman DrPH), University of Rajshahi, Rajshahi, Bangladesh; Institute of Health and Wellbeing (Prof M Rahman PhD), Federation University Australia, Berwick, VIC, Australia; School of Nursing and Midwifery (Prof M Rahman PhD), La Trobe University, Melbourne, VIC, Australia; Guilan Road Trauma Research Center (N Rahmanian PhD), Guilan University of Medical Sciences, Rasht, Iran; Health Service Research and Quality of Life Center (CEReSS) (Prof M Rahmati PhD), Aix-Marseille University, Marseille, France; Students Research Committee (R Rahmati MD), Shahrekord University of Medical Sciences, Shahrekord, Iran; Department of Cardiology (Prof M M Ramadan PhD), Faculty of Pharmacy (Prof M A Saleh PhD), Rheumatology and Immunology Unit (Prof S Tharwat MD), Mansoura University, Mansoura, Egypt; Independent Consultant, Triolet, Mauritius (K Ramphul MD); South Asian Institute for Social Transformation (SAIST), Dhaka, Bangladesh (J Rana MPH); Department of Epidemiology, Biostatistics and Occupational Health (J Rana MPH), McGill University, Montreal, Canada; Thrombosis Research Group (S Rashedi MD), Brigham and Women's Hospital, Harvard Medical School, Boston, MA, USA; Section of Pulmonary and Critical Care Medicine (N Ravikumar MD), University of Chicago, Chicago, IL, USA; Department of Primary Care and Public Health (Prof S Rawaf MD, C Tabche MSc), Imperial College London, London, UK; Academic Public Health England (Prof S Rawaf MD), Public Health England, London, UK; Department of Biological Sciences (Prof E M M Redwan PhD), King Abdulaziz University, Jeddah, Egypt; Department of Protein Research (Prof E M M Redwan PhD), Research and Academic Institution, Alexandria, Egypt; Department of Labour (P Roy PhD), Directorate of Factories, Government of West Bengal, Kolkata, India; Cardiovascular Department (Prof A M A Saad MD), Zagazig University, Zagazig, Egypt; Department of Pharmaceutical Chemistry (Prof M Saeb PhD), International Medical University, Gdańsk, Poland; Centre for Drug Design (N Y Sani PhD), Science University of Malaysia, Penang, Malaysia; Department of Pharmaceutical and Medicinal Chemistry (N Y Sani PhD), Bayer, Pulau Penang, Malaysia; Indira Gandhi Medical College and Research Institute, Puducherry, India (A Saravanan MD); Department of Medical Informatics (J Saulam MSc), Kagawa University, Miki-cho, Japan; Food Processing and Nutrition (J Saulam MSc), Karnataka State Akkamahadevi Women's University, Vijayapura, India; Department of Cardiovascular Sciences (A Schuermans BSc, J Van den Eynde BSc), Katholieke Universiteit Leuven, Leuven, Belgium; Department of Public Health (B Semagn MPH), Debre Berhan University, Debre Berhan, Ethiopia; Department of Medicine and Surgery (Y Sethi MBBS), Government Doon Medical College, Dehradun, India; National Heart, Lung, and Blood Institute (A Seylani BS), National Institute of Health, Rockville, MD, USA; Department of Safety Services (S Sharfaei MD), Baim Institute for Clinical Research, Boston, MA, USA; UN Mehta Institute of Cardiology and Research Center (Prof K Sharma MD), B.J. Medical College, Ahmedabad, India; Department of Cardiology (Prof K Sharma MD), Government Medical College, Ahmedabad, India; Independent Consultant, Worcester, MA, USA (N Sharma MD); Department of Cardiology (A A Sherif MD), McKenzie-Willamette Medical Center, Springfield, OR, USA; Oulu Business School (I Shiue PhD), Martti Ahtisaari Institute (I Shiue PhD),

University of Oulu, Oulu, Finland; Department of Medical-Surgical Nursing (S Shorofi PhD), Mazandaran University of Medical Sciences, Sari, Iran; Department of Medical Microbiology and Infectious Diseases (E E Siddig MD), Erasmus University, Rotterdam, Netherlands; Department of Pulmonary and Critical Care Medicine (H Singh MD), Medical College of Wisconsin, Milwaukee, WI, USA; School of Medicine (Prof J A Singh MD), Henry JN Taub Department of Emergency Medicine (Prof L Szarpak PhD), Baylor College of Medicine, Houston, TX, USA; Medicine Service (Prof J A Singh MD), US Department of Veterans Affairs (VA), Houston, TX, USA; Department of Radiodiagnosis (P Singh MD), Department of Community Medicine and Family Medicine (M Verma MD), All India Institute of Medical Sciences, Bathinda, India; Department of Health Education and Promotion (F Sobia PhD), Jazan University, Jazan, Saudi Arabia; Department of Systemic Pathology (R Solanki MD), Touro College of Osteopathic Medicine, Middletown, NY, USA; Department of Pathology (R Solanki MD), American University of the Caribbean School of Medicine, Cupecoy, Saint Martin; Department of Biochemistry (S Solanki MD), American University of Integrative Sciences, Bridgetown, Barbados; 3rd Department of Cardiology (M Spartalis PhD), University of Athens, Athens, Greece; Department of Analytical and Applied Economics (C Swain MPhil), Utkal University, Bhubaneswar, India; Department of Clinical Research and Development (Prof L Szarpak PhD), LUXMED Group, Warsaw, Poland; Department of Epidemiology (J L Tamuzi MSc), Stellenbosch University, Cape Town, South Africa; Department of Medicine (J L Tamuzi MSc), Northlands Medical Group, Omuthiya, Namibia; Department of Epidemiology and Biostatistics (M Teramoto MD), University of California San Francisco, San Francisco, CA, USA; Department of Medicine (Prof F Thienemann PhD), University of Cape Town, Cape Town, South Africa; Department of Internal Medicine (Prof F Thienemann PhD), University of Zürich, Zurich, Switzerland; Faculty of Medicine (T T Truyen MD), Nam Can Tho University, Can Tho, Vietnam; Department of Nursing (G M Tsegay MSc), Aksum University, Aksum, Ethiopia; Department of Biosciences and Biotechnology (A J Udoakang PhD), University of Medical Sciences, Ondo, Ondo, Nigeria; School of Public Health (F Wang PhD), Xuzhou Medical University, Xuzhou, China; Department of Community Medicine (N D Wickramasinghe MD), Rajarata University of Sri Lanka, Anuradhapura, Sri Lanka; Faculty of Health Sciences (A Wilandika MSc), Universitas Aisyiyah Bandung, Bandung, Indonesia; Department of Endocrinology (Prof S Xu PhD), University of Science and Technology of China, Hefei, China; School of Medicine (Prof S Xu PhD), University of Rochester, Rochester, NY, USA; Department of Epidemiology and Biostatistics (Prof C Yu PhD), School of Public Health (Prof Z Zhang PhD), Wuhan University, Wuhan, China; Research and Development Department (I Zare BSc), Sina Medical Biochemistry Technologies, Shiraz, Iran; Department of GI Medical Oncology (M A Zeineddine MD), University of Texas, Houston, FL, USA; The Institute of Cancer Research (L Zhu PhD), The Third Affiliated Hospital of Kunming Medical University, Kunming, China; Department of Biochemistry and Pharmacogenomics (M Zielińska MPharm), Medical University of Warsaw, Warsaw, Poland

Authors' Contributions

Managing the overall research enterprise

Catherine O Johnson, Christopher J L Murray, and Gregory A Roth.

Writing the first draft of the manuscript

Paul A Corris, Hillary M DuBrock, Sophia Emmons-Bell, Catherine O Johnson, Peter J Leary, Megan Lindstrom, Christopher J L Murray, Stuart Rich, Gregory A Roth, and Corey E Ventetuolo.

Primary responsibility for applying analytical methods to produce estimates

Sophia Emmons-Bell, Catherine O Johnson, Megan Lindstrom, Christopher J L Murray, and Gregory A Roth.

Primary responsibility for seeking, cataloguing, extracting, or cleaning data; designing or coding figures and tables

Sophia Emmons-Bell, Catherine O Johnson, and Megan Lindstrom.

Providing data or critical feedback on data sources

Yohannes Habtegiorgis Abate, Richard Gyan Aboagye, Salahdein Aburuz, Leticia Akua Adzigbli, Bright Opoku Ahinkorah, Ayman Ahmed, Haroon Ahmed, Salah Al Awaidy, Almaza Albakri, Awais Altaf, Hany Aly, Saleha Anwar, Ahmed Y. Azzam, Sara Bagherieh, Ovidiu Constantin Baltatu, Hiba Jawdat Barqawi, Mohammad-Mahdi Bastan, Nebiyou Simegnew Bayleyegn, Sonu Bhaskar, Vivek Bhat, Archith Boloor, Dejana Braithwaite, Michael Brauer, Lemma N Bulto, Vijay Kumar Chattu, Bryan Chong, Rajiv Chowdhury, Paul A Corris, Natalia Cruz-Martins, Xiaochen Dai, Sean DeAngelo, Berecha Hundessa Demessa, Hardik Dineshbhai Desai, Vishal R Dhulipala, Michael J Diaz, Thanh Chi Do, Thao Huynh Phuong Do, Khanh Duy Doan, Wendel Mombaque dos Santos, Rajkumar Prakashbhai Doshi, Sophia Emmons-Bell, Adeniyi Francis Fagbamigbe, Celia Fortuna Rodrigues, Mahaveer Golechha, Shi-Yang Guan, Simon I Hay, Mehdi Hemmati, Nguyen Quoc Hoan, Michael Hultström, Hong-Han Huynh, Segun Emmanuel Ibitoye, Olayinka Stephen Ilesanmi, Nahlah Elkudssiah Ismail, Khushleen Jaggi, Mihajlo Jakovljevic, Sun Ha Jee, Bijay Mukesh Jeswani, Anil K Jha, Catherine O Johnson, Jacek Jerzy Jozwiak, Pedram Keshavarz, Min Seo Kim, Shivakumar KM Marulasiddaiah Kondlahalli, Nikhil Kothari, Kewal Krishan, Thao Thi Thu Le, Peter J Leary, Caterina Ledda, Seung Won Lee, Stephen S Lim, Elham Mahmoudi, Kashish Malhotra, Deborah Carvalho Malta, Arup Kumar Misra, Ali H Mokdad, Mohammad Ali Moni, Rohith Motappa, Ahmad Mustafa, Ganesh R Naik, Dhairya P Nanavaty, Zuhair S Natto, Dang H Nguyen, Phat Tuan Nguyen, Robina Khan Niazi, Bogdan Oancea, Gláucia Maria Moraes Oliveira, Hany A Omar, Mahesh Padukudru P A, Romil R Parikh, Hoang Nhat Pham, Hoang Tran Pham, Anil K Philip, Elton Junio Sady Prates, Jagadeesh Puvvula, Fakher Rahim, Mohammad Rahmanian, Masoud Rahmati, Mahmoud Mohammed Ramadan, Juwel Rana, Sina Rashedi, Nakul Ravikumar, Salman Rawaf, Ayita Ray, Priyanka Roy, Aly M A Saad, Basema Ahmad Saddik, Ushasi Saraswati, Yashendra Sethi, Muhammad Aaqib Shamim, Anas Shamsi, Ivy Shiue, Jasvinder A Singh, Paramdeep Singh, Michael Spartalis, Chandan Kumar Swain, Lukasz Szarpak, Seyyed Mohammad Tabatabaei, Ker-Kan Tan, Friedrich Thienemann, Guesh Mebrahtom Tsegay, Jef Van den Eynde, Shoban Babu Varthya, Corey E Ventetuolo, Maria Viskadourou, Angga Wilandika, Suowen Xu, Chuanhua Yu, Iman Zare, Mohammad A Zeineddine, Zhi-Jiang Zhang, and Magdalena Zielińska.

Developing methods or computational machinery

Walid Adnan Al-Zyoud, Aleksandr Y Aravkin, Ahmed Y. Azzam, Mohammad-Mahdi Bastan, Sonu Bhaskar, Xiaochen Dai, Hardik Dineshbhai Desai, Thanh Chi Do, Robert Kokou Dowou, Sophia Emmons-Bell, Adeniyi Francis Fagbamigbe, Molla Getie, Shi-Yang Guan, Simon I Hay, Michael Hultström, Hong-Han Huynh, Chidozie Declan Iwu, Bijay Mukesh Jeswani, Catherine O Johnson, Thao Thi Thu Le, Peter J Leary, Megan Lindstrom, Elham Mahmoudi, Ali H Mokdad, Mohammad Ali Moni, Christopher J L Murray, Phat Tuan Nguyen, Hoang Tran Pham, Gregory A Roth, Austin E Schumacher, Yashendra Sethi, Michael Spartalis, and Chandan Kumar Swain.

Providing critical feedback on methods or results

Yohannes Habtegiorgis Abate, Michael Abdelmasseh, Richard Gyan Aboagye, Hasan Abualruz, Eman Abu-Gharbieh, Salahdein Aburuz, Lawan Hassan Adamu, Rui Adão, Isaac Yeboah Addo, Rufus Adesoji Adedoyin, Leticia Akua Adzigbli, Bright Opoku Ahinkorah, Firdos Ahmad, Amir Mahmoud Ahmadzade, Ayman Ahmed, Haroon Ahmed, Syed Anees Ahmed, Shiva Akhlaghi, Salah Al Awaidy, Samer O Alalalmeh, Almaza Albakri, Khalifah A Aldawsari, Wael Almahmeed, Najim Z Alshahrani, Awais Altaf, Hany Aly, Karem H Alzoubi, Walid Adnan Al-Zyoud, Reza Amani, Ganiyu Adeniyi Amusa, Catalina Liliana Andrei, Saleha Anwar, Anayochukwu Edward Anyasodor, Demelash Areda, Haftu Asmerom Asmerom, Avinash Aujayeb, Ahmed Y. Azzam, Abraham Samuel Babu, Sara Bagherieh, Ovidiu Constantin Baltatu, Hiba Jawdat Barqawi, Mohammad-Mahdi Bastan, Kavita Batra, Nebiyou Simegnew Bayleyegn, Amir Hossein Behnoush, Jaideep Singh Bhalla, Sonu Bhaskar, Vivek Bhat, Veera R Bitra, Archith Boloor, Dejana Braithwaite, Michael Brauer, Lemma N Bulto, Yasser Bustanji, Vijay Kumar Chattu, Gerald Chi, Bryan Chong, Rajiv Chowdhury, Zinhle Cindi, Paul A Corris, Natalia Cruz-Martins, Omid Dadras, Xiaochen Dai, Mohadese Dashtkoohi, Sean DeAngelo, Shayom Debopadhaya, Berecha Hundessa Demessa, Hardik Dineshbhai Desai, Vishal R Dhulipala, Michael J Diaz, Mengistie Diress, Thanh Chi Do, Thao Huynh Phuong Do, Khanh Duy Doan, Wendel Mombaque dos Santos, Rajkumar Prakashbhai Doshi, Robert Kokou Dowou, Hilary M DuBrock, Arkadiusz Marian Dziedzic, Muhammed Elhadi, Sophia Emmons-Bell, Farshid Etaee, Natalia Fabin, Adeniyi Francis Fagbamigbe, Pawan Sirwan Faris, Bikila Regassa Feyisa, Celia Fortuna Rodrigues, Aravind P Gandhi, Mohammad Arfat Ganiyani, Molla Getie, Amir Ghaffari Jolfayi, Afsaneh Ghasemzadeh, Mahaveer Golechha, Shi-Yang Guan, Mesay Dechasa Gudeta, Mohak Gupta, Rahul Gupta, Mostafa Hadei, Ahmad Hammoud, Md Saquib Hasnain, Mahgol Sadat Hassan Zadeh Tabatabaei, Simon I Hay, Mehdi Hemmati, Yuta Hiraike, Nguyen Quoc Hoan, Michael Hultström, Hong-Han Huynh, Segun Emmanuel Ibitoye, Olayinka Stephen Ilesanmi, Nahlah Elkudssiah Ismail, Chidozie Declan Iwu, Khushleen Jaggi, Akhil Jain, Mihajlo Jakovljevic, Sun Ha Jee, Bijay Mukesh Jeswani, Anil K Jha, Catherine O Johnson, Mohammad Jokar, Nitin Joseph, Jacek Jerzy Jozwiak, Hannaneh Kabir, Farima Kahe, Arun Kamireddy, Hanie Karimi, Arman Karimi Behnagh, Sina Kazemian, Pedram Keshavarz, Amirmohammad Khalaji, Mohammad Jobair Khan, Feriha Fatima Khidri, Min Seo Kim, Shivakumar KM Marulasiddaiah Kondlahalli, Nikhil Kothari, Kewal Krishan, Mukhtar Kulimbet, Ashish Kumar, Kaveh Latifinaibin, Thao Thi Thu Le, Peter J Leary, Caterina Ledda, Seung Won Lee, Ming-Chieh Li, Stephen S Lim, Megan Lindstrom, Shuke Liu, Elham Mahmoudi, Omar M Makram, Kashish Malhotra, Ahmad Azam Malik, Deborah Carvalho Malta, Yosef Manla, Miquel Martorell, Kamran Mehrabani-Zeinabad, Mohsen Merati, Tomislav Mestrovic, Arup Kumar Misra, Ali H Mokdad, Mohammad Ali Moni, AmirAli Moodi Ghalibaf, Paula Moraga, Negar Morovatdar, Rohith Motappa, Seyed Ali Mousavi-Aghdas, Ahmad Mustafa, Ganesh R Naik, Mohammad Sadeq Najafi, Soroush Najdaghi, Dhairya P Nanavaty, Delaram Narimani Davani, Zuhair S Natto, Javaid Nauman, Dang H Nguyen, Phat Tuan Nguyen, Robina Khan Niazi, Bogdan Oancea, Titilope O Olanipekun, Hany A Omar, Mahesh Padukudru P A, Feng Pan, Seithikurippu R Pandi-Perumal, Ioannis Pantazopoulos, Romil R Parikh, Ionela-Roxana Petcu, Hoang Nhat Pham, Hoang Tran Pham, Anil K Philip, Elton Junio Sady Prates, Jagadeesh Puvvula, Gangzhen Qian, Quinn Rafferty, Fakher Rahim, Mehran Rahimi, Mosiur Rahman, Muhammad Aziz Rahman, Mohammad Rahmanian, Nazanin Rahmanian, Masoud Rahmati, Rahem Rahmati, Mahmoud Mohammed Ramadan, Kamleshun Ramphul, Juwel Rana, Indu Ramachandra Rao, Sina Rashedi, Nakul Ravikumar, Salman Rawaf, Ayita Ray, Murali Mohan Rama Krishna Reddy, Elrashdy Moustafa Mohamed Redwan, Negar Rezaei, Stuart Rich, Aly M A Saad, Basema Ahmad Saddik, Mohammad Reza Saeb, Fatemeh Saheb Sharif-Askari, Narjes Saheb Sharif-Askari, Mohamed A Saleh, Najib Yahaya Sani, Ushasi Saraswati, Jennifer Saulam, Art

Schuermans, Birhan Ewunu Semagn, Yashendra Sethi, Melika Shafeghat, Moyad Jamal Shahwan, Muhammad Aaqib Shamim, Anas Shamsi, Sadaf Sharfaei, Nitish Sharma, Akil Adrian Sherif, Ivy Shiue, Seyed Afshin Shorofi, Emmanuel Edwar Siddig, Jasvinder A Singh, Paramdeep Singh, Farrukh Sobia, Michael Spartalis, Chandan Kumar Swain, Lukasz Szarpak, Seyyed Mohammad Tabatabaei, Celine Tabche, Jacques Lukenze Tamuzi, Ker-Kan Tan, Masayuki Teramoto, Samar Tharwat, Friedrich Thienemann, Guesh Mebrahtom Tsegay, Aniefiok John Udoakang, Jef Van den Eynde, Shoban Babu Varthya, Corey E Ventetuolo, Madhur Verma, Dominique Vervoort, Manish Vinayak, Maria Viskadourou, Fang Wang, Nuwan Darshana Wickramasinghe, Angga Wilandika, Suowen Xu, Chuanhua Yu, Mohammad A Zeineddine, Zhi-Jiang Zhang, Lei Zhu, Magdalena Zielińska, and Samer H Zyoud.

Drafting the work or revising it critically for important intellectual content

Yohannes Habtegiorgis Abate, Michael Abdelmasseh, Hasan Abualruz, Eman Abu-Gharbieh, Salahdein Aburuz, Lawan Hassan Adamu, Rui Adão, Isaac Yeboah Addo, Rufus Adesoji Adedoyin, Juliana Bunmi Adetunji, Bright Opoku Ahinkorah, Ayman Ahmed, Haroon Ahmed, Syed Anees Ahmed, Mohammed Ahmed Akkaif, Salah Al Awaidy, Samer O Alalalmeh, Almaza Albakri, Wael Almahmeed, Najim Z Alshahrani, Awais Altaf, Hany Aly, Karem H Alzoubi, Walid Adnan Al-Zyoud, Reza Amani, Ganiyu Adeniyi Amusa, Catalina Liliana Andrei, Saleha Anwar, Anayochukwu Edward Anyasodor, Haftu Asmerom Asmerom, Avinash Aujayeb, Ahmed Y. Azzam, Abraham Samuel Babu, Sara Bagherieh, Ovidiu Constantin Baltatu, Hiba Jawdat Barqawi, Mohammad-Mahdi Bastan, Amir Hossein Behnoush, Jaideep Singh Bhalla, Sonu Bhaskar, Vivek Bhat, Saeid Bitaraf, Veera R Bitra, Dejana Braithwaite, Yasser Bustanji, Vijay Kumar Chattu, Fatemeh Chichagi, Bryan Chong, Rajiv Chowdhury, Paul A Corris, Natalia Cruz-Martins, Sriharsha Dadana, Tukur Dahiru, Mohadese Dashtkoohi, Berecha Hundessa Demessa, Hardik Dineshbhai Desai, Vishal R Dhulipala, Michael J Diaz, Thanh Chi Do, Khanh Duy Doan, Wendel Mombaque dos Santos, Rajkumar Prakashbhai Doshi, Robert Kokou Dowou, Hilary M DuBrock, Arkadiusz Marian Dziedzic, Muhammed Elhadi, Natalia Fabin, Adeniyi Francis Fagbamigbe, Mohammad Arfat Ganiyani, Yibeltal Yismaw Gela, Afsaneh Ghasemzadeh, Mohamad Goldust, Shi-Yang Guan, Mesay Dechasa Gudeta, Mohak Gupta, Rahul Gupta, Mostafa Hadei, Ahmad Hammoud, Md Saquib Hasnain, Mahgol Sadat Hassan Zadeh Tabatabaei, Simon I Hay, Omar E Hegazi, Mehdi Hemmati, Yuta Hiraike, Nguyen Quoc Hoan, Michael Hultström, Hong-Han Huynh, Segun Emmanuel Ibitoye, Olayinka Stephen Ilesanmi, Nahlah Elkudssiah Ismail, Chidozie Declan Iwu, Khushleen Jaggi, Akhil Jain, Mihajlo Jakovljevic, Bijay Mukesh Jeswani, Catherine O Johnson, Nitin Joseph, Jacek Jerzy Jozwiak, Farima Kahe, Arun Kamireddy, Arun R Kanmanthareddy, Hanie Karimi, Amirmohammad Khalaji, Mohammad Jobair Khan, Feriha Fatima Khidri, Min Seo Kim, Shivakumar KM Marulasiddaiah Kondlahalli, Kewal Krishan, Mukhtar Kulimbet, Kaveh Latifinaibin, Thao Thi Thu Le, Peter J Leary, Caterina Ledda, Megan Lindstrom, Elham Mahmoudi, Omar M Makram, Kashish Malhotra, Ahmad Azam Malik, Deborah Carvalho Malta, Miquel Martorell, Kamran Mehrabani-Zeinabad, Mohsen Merati, Tomislav Mestrovic, Niloofar Mirdamadi, Ali H Mokdad, Mohammad Ali Moni, AmirAli Moodi Ghalibaf, Paula Moraga, Rohith Motappa, Seyed Ali Mousavi-Aghdas, Christopher J L Murray, Ahmad Mustafa, Mohammad Sadeq Najafi, Soroush Najdaghi, Delaram Narimani Davani, Zuhair S Natto, Javaid Nauman, Dang H Nguyen, Phat Tuan Nguyen, Robina Khan Niazi, Bogdan Oancea, Titilope O Olanipekun, Hany A Omar, Mahesh Padukudru P A, Feng Pan, Ioannis Pantazopoulos, Romil R Parikh, Ionela-Roxana Petcu, Hoang Nhat Pham, Hoang Tran Pham, Anil K Philip, Elton Junio Sady Prates, Fakher Rahim, Mehran Rahimi, Mohammad Rahmanian, Rahem Rahmati, Mahmoud Mohammed Ramadan, Sina Rashedi, Nakul Ravikumar, Salman Rawaf, Ayita Ray, Elrashdy Moustafa Mohamed Redwan, Stuart Rich, Gregory A Roth, Priyanka Roy, Aly M A Saad, Basema Ahmad Saddik, Masoumeh Sadeghi, Fatemeh Saheb Sharif-Askari, Narjes Saheb Sharif-Askari, Najib Yahaya Sani,

Aswini Saravanan, Art Schuermans, Yashendra Sethi, Allen Seylani, Moyad Jamal Shahwan, Muhammad Aaqib Shamim, Anas Shamsi, Kamal Sharma, Nitish Sharma, Akil Adrian Sherif, Seyed Afshin Shorofi, Emmanuel Edwar Siddig, Harpreet Singh, Jasvinder A Singh, Paramdeep Singh, Surjit Singh, Farrukh Sobia, Ranjan Solanki, Shipra Solanki, Michael Spartalis, Chandan Kumar Swain, Lukasz Szarpak, Celine Tabche, Jacques Lukenze Tamuzi, Ker-Kan Tan, Masayuki Teramoto, Samar Tharwat, Friedrich Thienemann, Thien Tan Tri Tai Truyen, Aniefiok John Udoakang, Jef Van den Eynde, Shoban Babu Varthya, Corey E Ventetuolo, Madhur Verma, Dominique Vervoort, Manish Vinayak, Fang Wang, Nuwan Darshana Wickramasinghe, Iman Zare, Mohammad A Zeineddine, Abzal Zhumagaliuly, Magdalena Zielińska, and Samer H Zyoud.

Managing the estimation or publications process

Simon I Hay, Catherine O Johnson, Peter J Leary, Ali H Mokdad, Christopher J L Murray, and Gregory A Roth.
